# Entry and price competition in the over‐the‐counter drug market after deregulation: Evidence from Portugal

**DOI:** 10.1002/hec.4109

**Published:** 2020-06-08

**Authors:** Ana Moura, Pedro Pita Barros

**Affiliations:** ^1^ Department of Econometrics and Operations Research Tilburg University Tilburg Netherlands; ^2^ Nova School of Business and Economics Universidade Nova de Lisboa Lisbon Portugal

**Keywords:** market liberalization, over‐the‐counter drugs, pharmaceutical market, price competition

## Abstract

In the last two decades, many European countries allowed the sale of over‐the‐counter (OTC) drugs outside pharmacies. This was expected to lower retail prices through increased competition. Evidence of such price reductions is scarce. We assess the impact of supermarket and outlet entry in the OTC drug market on OTC prices charged by incumbent pharmacies using a difference‐in‐differences strategy. We use price data on five popular OTC drugs for all retailers located in Lisbon for three distinct points in time (2006, 2010, and 2015). Our results suggest that competitive pressure in the market is mainly exerted by supermarkets, which charge, on average, 20% lower prices than pharmacies. The entry of a supermarket among the main competitors of an incumbent pharmacy is associated with an average 4% to 6% decrease in prices relative to the control group. These price reductions are long‐lasting but fairly localized. We find no evidence of price reductions following OTC outlet entry. Additional results from a reduced‐form entry model and a propensity score matching difference‐in‐differences approach support the view that these effects are causal.

## 1 INTRODUCTION

Over‐the‐counter (OTC) drugs are pharmaceuticals whose purchase does not require a prescription. They are usually not reimbursed and their pricing is free, in contrast with the highly regulated prices of reimbursed and/or prescription‐only pharmaceuticals.

During the last two decades, European countries have extensively reformed their community pharmacy sectors. An important element of these reforms is the liberalization of OTC medicine distribution OECD (2014). OTC market liberalization implies a move from a traditional pharmacy‐centered model to a multichannel distribution model in which OTC drugs are sold outside pharmacies, namely, in supermarkets, petrol stations, and other nonpharmacy outlets. Throughout this paper, we refer to these as nonpharmacy retailers.

The rationale for OTC market liberalization was that the entry of nonpharmacy retailers, combined with free OTC pricing, would lower OTC drug prices via increased competition among retailers (Lluch & Kanavos, 2010; Stargardt et al. 2007; Morgall & Almarsdóttir, 1999). Existing literature posits that pharmacies are not used to price competition and do not place competitive constraints on each other (Pilorge, 2016; Stargardt et al. 2007). 1This inability may be associated with either the development of close professional relationships among pharmacists or their use to the noncompetitive environment in place prior to market liberalization. Alternatively, pharmacies may not compete in prices but rather in quality, range of services, location, or opening times (Martins & Queirós, 2015; Lluch & Kanavos, 2010; Anell, 2005; Rudholm, 2008; Stargardt et al. 2007; Schaumans & Verboven, 2008).The fact that, at least in urban areas, nonpharmacy retailers charge lower prices than traditional pharmacies (Anell, 2005; OFT, 2003) might mechanically lead to lower average prices but provides no evidence of competitive forces. We examine whether facing increased competitive pressure following the entry of a nonpharmacy competitor, who is able to charge lower prices, triggers price decreases by incumbent pharmacies.

This is an important question that, to the best of our knowledge, has not yet been fully addressed in the literature. OTC drugs are one of the few product segments for which pharmacies can make their own pricing decisions. Because they are frequently used, we expect consumers to be aware of price differences between retailers (Sorensen, 2000). By shedding light on how competition takes place in this market, we contribute to inform policy makers on the market dynamics they might expect upon liberalizing OTC medicine distribution. The empirical analysis draws on the Portuguese experience. In Portugal, OTC market liberalization started in late 2005 and allowed OTC drugs to be sold outside pharmacies, namely, in supermarkets and outlets. 2Patrício et al. (2005), Centro de Estudos de Gestão e Economia Aplicada (CEGEA) (2005), and Gomes (2007) used the classic frameworks of Hotelling (1929) and Waterson (1993) to make predictions of the expected price outcomes of the reform. These predictions pointed in very different directions, and the real impact of the reform was never assessed.OTC market liberalization reforms similar to the Portuguese one were implemented all over Europe during the last two decades: in 2000, Poland allowed for a limited range of OTC products to be sold outside pharmacies; Denmark, Norway, Italy, Hungary, Sweden, and France adopted similar policies in the following years; Germany and the United Kingdom had already done so during the 1990s.

We use price data for five popular OTC drugs across all retailer types (traditional pharmacies, supermarkets, and outlets) located in Lisbon. The dataset has a panel structure, and each retailer is observed for at most three points in time, the years of 2006, 2010, and 2015. In our data, supermarkets and outlets charge, on average, 20% and 4% lower prices than traditional pharmacies, respectively.

Our empirical strategy is a difference‐in‐differences (DID) design, comparing the prices charged by pharmacies that experience entry of a supermarket or outlet among their main competitors and the prices charged by pharmacies that do not experience entry of a supermarket or outlet among their main competitors, before and after entry occurs. We use two alternative baseline measures to define the set of main competitors of a pharmacy. One measure takes as main competitors of a pharmacy its three nearest neighbors selling OTC drugs. The other measure takes as main competitors of a pharmacy all retailers located within a 400‐m radius distance. Identification comes from the different timing of exposure of incumbent pharmacies to different types of nonpharmacy entrants among their main competitors.

Our main results show that incumbent pharmacies lower their prices by about 6% after experiencing the entry of a supermarket among their three nearest neighbors. We do not find evidence that outlet entry leads to price reductions by pharmacies. We find a fair degree of heterogeneity in price responses across pharmacies operating in areas with different degrees of market concentration with our results being driven by the most isolated pharmacies, who likely enjoyed some degree of market power prior to experiencing entry. We obtain similar results when using a 400‐m radius to define the set of main competitors of a pharmacy. We interpret our findings in the context of a model based on Salop (1979) with nonpharmacy entrants differing from incumbent pharmacies in their marginal cost and, in particular, supermarkets being more efficient.

Our results do not seem to be driven by existing pretreatment trends and survive a battery of robustness checks. When varying the number of nearest neighbors and the radius distance that define the set of main competitors of a pharmacy, we find that the statistical significance of our results falls quickly as we enlarge the set of main competitors of a pharmacy, suggesting competition is fairly localized. The causal interpretation of our findings, however, rests on the assumption that market structure is exogenous so that exposure to nonpharmacy entry is random. We address endogeneity concerns in two ways. First, we implement a propensity score matching DID approach, with propensity scores being a function of pre‐entry levels of competitive pressure and demand faced by each pharmacy, and obtain results that are broadly in line with our main findings, although less statistically significant. Second, we estimate a reduced‐form entry model in which the probability that a pharmacy faces nonpharmacy entry is a function of past prices. We find no evidence of an association between past prices and nonpharmacy entry.

Our findings contribute to the empirical literature on OTC drug pricing and the effects of OTC market liberalization in Europe. This literature is scarce, mostly descriptive, and often unable to confirm the expected downward trend in OTC prices (OECD, 2014; Vogler et al. 2014). We show that OTC liberalization reforms can lower prices via increased competition, though this crucially depends on the ability of entrants to exert competitive pressure on incumbent pharmacies.

Our study also contributes to a broader literature within industrial organization on the price effects following the entry of supermarkets and chain stores in general in a market previously composed of small, independent firms, as is the case of traditional pharmacies in Portugal. 3Traditional pharmacies in Portugal are independently owned because of existing ownership restrictions that limit the number of pharmacies that an agent can own. Ownership restrictions are common and seek to ensure a certain degree of market competition. Recently, organized groups of independently‐owned pharmacies were created, but our data are prior to that.Bennett and Yin (2019) study the entry of a retail pharmacy chain in India on the price of incumbent pharmacies. Basker (2005) studies the effect of Walmart entry on average city‐level prices, and Basker and Noel (2009) estimate its effects on competitors' prices. We contribute to this literature by providing evidence for the OTC drug market.

The remainder of this paper is as follows. Section 2 provides institutional background on the Portuguese OTC market and the liberalization process. Section 3 describes the dataset, and Section 4 presents the empirical strategy. Section 5 presents the results, and Section 6 concludes.

## 2 INSTITUTIONAL BACKGROUND

Traditionally, community pharmacies enjoyed a monopoly for selling both prescription and OTC drugs. In Portugal, their monopoly for selling OTC drugs ended with Decree‐Law n. 134/2005 (August 16, 2005), which allowed the sale of OTC drugs outside pharmacies. 4The Portuguese government announced the intention to liberalize the OTC market a few months before Decree‐Law 134/2005 was passed. We cannot completely rule out that pharmacies adopted strategies other than pricing to prevent nonpharmacy entry. Nevertheless, the fact that nonpharmacy entry took off quickly after liberalization, combined with pharmacies not being used to operate in a competitive environment, leaves less scope for such strategic behavior.Prescription drugs remain available only at traditional pharmacies.

The first nonpharmacy retailers entered the OTC market in October 2005. Nonpharmacy retailers can be of two types: supermarkets and outlets (*parafarmácias*).

In supermarkets, by regulation, OTC drugs are not are freely accessible to customers. They are placed either in a closed shelf located behind the cashiers' check‐out counter or in a dedicated area together with other wellness products. Either way, customers wishing to purchase a given OTC drug must request it from the cashier or the employee attending to the dedicated area. Most supermarkets selling OTC drugs in Lisbon belong to either one of the two biggest supermarket chains in Portugal.

Nonpharmacy outlets are stores selling cosmetics, baby care products, vitamins, and supplements, among others. OTC drugs represented a natural expansion of their product range. Outlets can be either independently owned or part of small chains of two or three stores.

Nonpharmacy retailers wishing to enter the Portuguese OTC market must apply for a license at the National Authority of Medicines and Health Products (Infarmed) and satisfy specific requirements related to drug storage, qualification of personnel, etc. Application by supermarket and outlet chains is done individually by each store belonging to the chain as opposed to one license application for all stores belonging to the chain.

The entry of supermarkets and outlets in the OTC market took place quickly following market liberalization. 5Throughout the paper, entry in the OTC market refers to the moment at which a retailer is granted a license to sell OTC drugs.In the first quarter of 2009, there were over 800 nonpharmacies in Portugal, and by the end of 2017, there were about 1,200. The volume share of OTC drugs in the total outpatient pharmaceutical market was 16.5% by the end of 2017. The corresponding value share was 11.7%. The nonpharmacy volume share of the OTC sector in Portugal has risen continuously since market liberalization, plateauing at 20% in 2014 (Infarmed 2018).

## 3 DATA

Our data consist of the prices of five popular OTC drugs charged by all pharmacies, supermarkets, and outlets located in the municipality of Lisbon for three different points in time, the years of 2006, 2010, and 2015.

The five OTC drugs are Aspirina 500 mg (20 pills, Bayer), Cêgripe (20 pills, Jassen‐Cilag Ltd.), Trifene200 (20 pills, Medinfar), Mebocaína Forte (20 tablets, Novartis), and Tantum Verde (mouthwash, Angelini). These drugs tackle simple conditions such as fever and headaches (Aspirina), colds (Cêgripe), menstrual pain (Trifene200), sore throat (Mebocaína Forte), and toothache and gum swelling (Tantum Verde). They are among the top‐selling OTC drugs in Portugal. In 2009, these five drugs accounted for 10.8% of the volume sales of OTC drugs outside pharmacies. All of them featured in the Top 15 best‐selling drugs in volume, and three of them featured in the Top 10 (Infarmed, 2010). 6After 2009, Infarmed stopped releasing sales data by commercial designation, so we do not have more recent figures. They are well‐known brands to consumers and often advertised in the media. More importantly, they are available at all retailers. 7Supermarkets and outlets typically carry a smaller selection of OTC drugs than pharmacies.


Price data for 2006 were kindly provided by Simões et al. (2006), who collected them between March and April. We then carried out two additional rounds of data collection, in 2010 and 2015. Infarmed keeps an online, updated list of all active retailers that are licensed to sell OTC drugs. We examined these lists before each data collection round and identified the active retailers and their exact locations. We collected price data for 2010 and 2015 between December 2010 and February 2011 and between February and April 2015, respectively.

Though Simões et al. (2006) visited every OTC retailer in 2006, some retailers were not willing to disclose price information, resulting in some missing price data for that year. When we carried out the data collection in 2010 and 2015, we purchased the drugs at retailers whose staff refused to disclose prices. In these two periods, we observe prices for all retailers located in Lisbon.

We use the latitude and longitude coordinates of each retailer to identify its main competitors at each time period. We also construct indicators for retailer type (traditional pharmacy, supermarket, or outlet) and the parish where each retailer is located. 8Portuguese municipalities are composed of smaller areas called parishes. The number and geographic borders of the Lisbon parishes were revised in 2012. According to the revised version, which we use in our analysis, there are 24 parishes in Lisbon.Finally, we have data from the 2001 Portuguese census on the population living in the census block where each retailer is located.

We follow retailers over the three time periods for which we have data. Our dataset is unbalanced because there are retailers entering and exiting the market between each data collection round. Appendix S2 shows maps of the OTC market structure in Lisbon for the years 2006, 2010, and 2015. The number of supermarkets selling OTC drugs in our dataset increased over time, from one in 2006 to 25 in 2015. The number of outlets selling OTC drugs raised from eight in 2006 to 25 in 2010 and then slightly declined to 21 in 2015. The number of traditional pharmacies has been declining over time, from 301 in 2006 to 259 in 2015.

We now highlight a few patterns present in our data. The average prices of the drugs under analysis increased over time, as did their variance. All supermarkets in our data belong to supermarket chains, and each chain adopts a common pricing strategy, rather than store‐specific prices that reflect the competitive environment faced by each store belonging to the chain. On average, supermarkets charge about 20% lower prices than traditional pharmacies for the sample of OTC drugs we analyze. This might be due to economies of scale in the distribution chain of supermarkets, more efficient practices regarding stock management and logistics, and stronger bargaining position when engaging in price negotiations with suppliers because of larger quantities purchased. All of these result, cumulatively, in lower marginal costs, leading to lower equilibrium prices for supermarkets. Outlet prices are, on average, 4% lower than those of traditional pharmacies. Outlets are either independent stores or part of very small chains, which might imply that they face wholesale prices similar to those faced by traditional pharmacies.

## 4 METHODOLOGY

### 4.1 Empirical strategy

We use a DID strategy to assess the price effects following the entry of nonpharmacy retailers. Nonpharmacy entry started before our first round of data collection. However, nonpharmacy entry took place gradually, meaning that each pharmacy experiences entry of different types of nonpharmacies among its main competitors at different points in time. This is our source of identification.

We start by defining the set of main competitors of pharmacy *i*. One way to define the main competitors of a pharmacy is to consider its *N* nearest neighbors in terms of walking distance as main competitors. 9We use walking distances instead of straight‐line distances to define the nearest competitors of each pharmacy. This accounts for physical barriers that might cause two nearby retailers not to be regarded as competitors by consumers, for example. a high‐speed road. We measured walking distances between retailers after each data collection round because they can change over time due to urban development; for example, a new aerial bridge might be built, allowing consumers to easily cross over a high‐speed road.Another way to define the main competitors of pharmacy *i* is to consider all retailers located within a radius *R* centered around *i* as main competitors. We use these two alternative definitions of main competitors throughout our analysis.

An incumbent pharmacy is “treated” if it experiences the entry of a nonpharmacy retailer among its main competitors. Prior to treatment, its set of main competitors consists only of traditional pharmacies. Because supermarkets and outlets charge different prices, they might exert different levels of competitive pressure on incumbent pharmacies and generate different price effects. We therefore distinguish two types of treatment, *SUPER*_*i*_ and *OUTLET*_*i*_, depending on whether the nonpharmacy entrant faced by pharmacy *i* is a supermarket or an outlet, respectively. Additionally, for each type of treatment, we distinguish three treatment cohorts, *c*, according to treatment timing. Each of the two types of treatment can take place either before 2006 (*c*=1, the first and earliest treatment cohort), between 2006 and 2010 (*c*=2, the second treatment cohort), or between 2010 and 2015 (*c*=3, the third and latest treatment cohort). In total, there are six treatment groups corresponding to two types of treatment and three treatment cohorts. The control group is composed of pharmacies who never face nonpharmacies among their main competitors.

We estimate the price differences between each treatment group and the control group at each of our sample years. The regression counterpart of these differences is as follows:

(1) $$\begin{array}{ccc} P_{ikt} & =\theta_{ct}^{super}\times \delta_{t}SUPER_{i}^{c}+\theta_{ct}^{outlet}\times \delta_{t}OUTLET_{i}^{c} & \\ & +\delta_{t}+\gamma_{k}+\alpha_{i}+\varepsilon_{ikt}, \end{array}$$

$$\begin{array}{ccc} \text{with}\;SUPER_{i}^{c} & =\{SUPER_{i}^{1},SUPER_{i}^{2},SUPER_{i}^{3}\} & \\ \text{and}\;OUTLET_{i}^{c} & =\{OUTLET_{i}^{1},OUTLET_{i}^{2},OUTLET_{i}^{3}\}. \end{array}$$

In Equation (1), *i* indexes the pharmacy, *t* indexes time in years, *k* indexes the drug, and *c* indexes the treatment cohort. The dependent variable is the natural logarithm of the price charged by pharmacy *i*, for drug *k* in year *t*. $SUPER_{i}^{c}$ and $OUTLET_{i}^{c}$ are vectors of indicators for each of the three cohorts that experienced the entry of a supermarket and outlet, respectively, among their main competitors. 10We use indicator variables for facing nonpharmacy entry, as opposed to measures of the general level of competitive pressure faced by a pharmacy. This is because we are specifically interested on the additional competitive pressure originating from the entry of different types of retailers, supermarkets, and outlets. Our main interest is not on the general level of competitive pressure originating from a higher density of traditional pharmacies in an area, which has been assessed in previous literature (see, for example, Pilorge, 2016). For example, $SUPER_{i}^{2}$ is a binary indicator taking value 1 in case pharmacy *i* experienced the entry of a supermarket among its main competitors between 2006 and 2010 (the second treatment cohort) and value 0 otherwise. Similarly, $OUTLET_{i}^{3}$ is a binary indicator taking value 1 if pharmacy *i* experienced the entry of an outlet among its main competitors between 2010 and 2015 (the third treatment cohort). *δ*_*t*_ is a vector containing fixed‐effects for years 2010 and 2015. *γ*_*k*_ and *α*_*i*_ are drug and retailer fixed‐effects, respectively. *ε*_*ikt*_ is an error term.

The main coefficients of interest are $\theta_{ct}^{super}$ and $\theta_{ct}^{outlet}$, corresponding to interactions between the treatment groups and year fixed‐effects. Their estimates convey the price impact of nonpharmacy entry on incumbent pharmacies and their dynamics over time. To be more precise, $\theta_{ct}^{super}$ conveys the price difference in year *t* between pharmacies that experience entry of a supermarket among their main competitors in treatment cohort *c* and pharmacies in the control group. An analogous interpretation applies to $\theta_{ct}^{outlet}$ for outlet entry.

Our empirical design is as flexible as possible, given that we only have data for three time points in time. Pharmacies experiencing supermarket and outlet entry after 2010 are observed twice prior to treatment, in 2006 and in 2010. The estimates of $\theta_{3,2010}^{super}$ and $\theta_{3,2010}^{outlet}$ correspond to price differences in 2010 between these pharmacies and the control group. Because these are price differences prior to treatment, the statistical significance of these estimates is informative about the plausibility of the parallel trend assumption.

Additionally, pharmacies experiencing supermarket and outlet entry between 2006 and 2010 are observed twice after treatment, in 2010 and in 2015. The estimates of $\theta_{2,2015}^{super}$ and $\theta_{2,2015}^{outlet}$ correspond to price differences in 2015 between these pharmacies and the control group. $\theta_{2,2010}^{super}$ and $\theta_{2,2010}^{outlet}$, in turn, convey a more immediate price impact of nonpharmacy entry on these pharmacies because they reflect price differences relative to the control group in 2010. Comparing these two pairs of estimates allows us to assess the persistence of the price effects induced by nonpharmacy entry.

When taking our model to the data, we select specific values of *N* and *R*. We set *N*=3 for our baseline nearest neighbor specification. In this case, the treatments consist on the entry of a supermarket or outlet in the set of three nearest neighbors before 2006, between 2006 and 2010, or between 2010 and 2015. We set *R*=400 m for our baseline radius specification. Under this definition, the treatments consist on the entry of a supermarket or outlet within a 400‐m radius before 2006, between 2006 and 2010, or between 2010 and 2015. We vary our choices of *N* and *R* in robustness checks.

We estimate Equation (1) using fixed‐effects at the pharmacy level, thus differencing out all time‐invariant, pharmacy‐specific characteristics. 11Our results are similar when using a random effects specification with parish and treatment group fixed‐effects (Table S3.1 in Appendix S3). From a statistical viewpoint, the random‐effects model assumes the constant retailer‐specific terms *α*_*i*_ to be independent drawings from an underlying population of retailers (see, for example, Heij et al. 2004). Because our data contain the universe of retailers operating in Lisbon, this assumption seems less appropriate in our case. A Hausman test also favors the fixed‐effects specification, so we use it throughout our analysis.We cluster standard errors at the pharmacy level to account for serial correlation in pharmacy pricing decisions. 12This clustering option is common when defining markets around a focal retailer (see Hosken et al. 2008) because in such settings, retailers are the relevant unit at which treatment assignment occurs. This is also in line with the recommendations of Abadie et al. (2017) to cluster standard errors at the level of treatment variation. We experimented with alternative ways of clustering the standard errors, namely two‐way clustering by pharmacy and drug. This does not affect the significance of our results (Table 3.9 in Appendix S3).


Because our main interest is on the effects on the pricing of incumbent pharmacies, we estimate our baseline model among pharmacies only. Throughout most of our analysis, we focus on samples in which all treatment and control groups are mutually exclusive. 13Pharmacies experiencing nonpharmacy entry at several points in time or experiencing both supermarket and outlet entry are disregarded from most of our analysis. This avoids having many interaction terms in the model whose identification relies on very few pharmacies, and it simplifies the construction of the propensity‐score‐matched sample. Our results are unchanged if we include pharmacies that experienced multiple treatments (Table 3.9 in Appendix S3).Thus, the number of pharmacies used in the estimation and the number of pharmacies in the treatment and control groups vary with the definition of main competitors. Table 1 shows the composition of the treatment and control groups for our baseline choices of main competitors: the three nearest neighbors and the retailers located within a 400‐m radius. 14Table 4.1 in Appendix S4 shows the composition of control and treatment groups for different choices of *N* and *R* used in robustness checks.In the specific case of the 400‐m radius measure, no pharmacies experienced entry of a supermarket before 2006, so that treatment group is empty. Each pharmacy belongs to the same group throughout all time periods in which it is observed. However, the number of pharmacies in each group can vary over time because of market entry and exit. For example, Table 1 conveys that some of the pharmacies that experienced entry of an outlet within a 400‐m radius exited the market. The increase in the number of pharmacies in the control group between 2006 and 2010 reflects the missing price data for 2006, as discussed in Section 3.

**TABLE 1:** Composition of control and treatment groups in the baseline sample

	Definition of main competitors					
	Three nearest neighbors			400‐m radius		
	2006	2010	2015	2006	2010	2015
Control group	152	220	197	136	206	186
Supermarket entry before 2006	2	2	2	0	0	0
Supermarket entry in 2006/10	6	6	6	5	5	5
Supermarket entry in 2010/15	13	13	13	6	6	6
Outlet entry before 2006	8	8	8	9	7	6
Outlet entry in 2006/10	10	10	10	14	14	12
Outlet entry in 2010/15	11	11	11	12	12	12
Total	202	270	247	182	250	227

^*Note*^: The table shows the number of pharmacies included in the baseline estimation samples per treatment group and year for our two alternative definitions of main competitors. In the first three columns, the main competitors of a pharmacy are defined as its three nearest neighbors. In the last three columns, the main competitors of a pharmacy are defined as all retailers located within a 400‐m radius. The lower number of pharmacies in the control group in 2006 is a consequence of missing price data for that year, as discussed in Section 3. Within a definition of main competitors, we focus on a sample of pharmacies for which all the treatment groups and the control group are mutually exclusive.

One concern is that pharmacies in the control group and those that eventually face nonpharmacy entry are already somewhat different prior to treatment. In Table 2, we compare the pretreatment means of our main variables for pharmacies in the control group and those treated after 2006. We do this for our two alternative measures of main competitors. We exclude pharmacies who experienced nonpharmacy entry before 2006, as for these we have no pretreatment observations. Pharmacies that eventually experience nonpharmacy entry charge lower prices for some of the drugs under analysis in 2006, and they tend to be located in areas with higher population. This motivates the estimation of Equation (1) on a matched sample of pharmacies (Section 4.2). 15Table 4.2 in Appendix S4 replicates this exercise for alternative treatment group definitions on the basis of different choices of *N* and *R* used in robustness checks.


**TABLE 2:** Testing for mean differences between groups of pharmacies at baseline (2006)

	Control group	Eventually treated	Difference	*p* value
*Main competitors: three nearest neighbors*				
Price *Aspirina 500 mg* (€)	3.033	2.996	0.026	0.378
Price *Cêgripe* (€)	4.312	4.195	0.118^∗∗∗^	0.002
Price *Trifene200* (€)	3.348	3.255	0.093^∗∗^	0.041
Price *Mebocaína Forte* (€)	4.676	4.613	0.062	0.238
Price *Tantum Verde* (€)	4.987	4.848	0.139^∗∗^	0.047
Avg distance to 3 nearest neighbors (km)	0.241	0.274	−0.035	0.241
Avg walking time to 3 nearest neighbors (min)	5.161	5.800	−0.639	0.323
Population in census block (as of 2001)	589.024	723.286	−125.262^∗∗∗^	0.002
*Main competitors: 400‐m radius*				
Price *Aspirina 500 mg* (€)	3.026	3.004	0.022	0.554
Price *Cêgripe* (€)	4.323	4.240	0.083^∗∗^	0.027
Price *Trifene200* (€)	3.340	3.285	0.055	0.149
Price *Mebocaína Forte* (€)	4.675	4.688	−0.013	0.213
Price *Tantum Verde* (€)	4.995	4.913	0.082	0.418
Number of retailers within radius	4.940	4.838	0.102	0.864
Population in census block (as of 2001)	590.694	646.255	−55.561	0.104

^*Note*^: The table shows the 2006 mean of several variables of interest across pharmacies in the control and treatment groups for our two alternative measures of main competitors. In the top panel, the main competitors of a pharmacy are its three nearest neighbors, and in the bottom panel, they are all retailers located within a 400‐m radius. For each panel, the first column reports averages across pharmacies belonging to the control group. The second column reports averages across pharmacies that were not yet treated in 2006 but will eventually face the entry of a nonpharmacy among their three nearest competitors, thus grouping together pharmacies facing the entry of a supermarket or an outlet either between 2006 and 2010 or between 2010 and 2015. Pharmacies already treated in 2006 are not accounted for in this table because they are not observed prior to treatment. The third column computes the difference of Columns 1 and 2, and Column 4 shows the corresponding two‐sided *p* value.

^*^ *p*<0.1.

^**^ *p*<0.05.

^***^ *p*<0.01.

Entry is expected to have stronger effects in areas where market structure is more concentrated, that is, closer to a monopoly. We assess this hypothesis by estimating Equation (1) among the most and the least spatially isolated pharmacies, alternatively. We define the most spatially isolated pharmacies on the basis of information for 2006. In the case of our nearest neighbors measure of main competitors, the most (least) spatially isolated pharmacies are those whose walking time in minutes to their third nearest competitor is above (below) the sample median in 2006. For our radius measure of competition, the most (least) spatially isolated pharmacies are those whose number of competitors within a 400‐m radius in 2006 is below (above) the sample median.

Our control group may be contaminated by second‐order effects related to the entry of nonpharmacies. That is, if Pharmacy A experiences the entry of Nonpharmacy B among its main competitors, A might lower its price (first‐order effect). That may cause C, who is in the control group and has A but not B among its main competitors, to change its price as a response to the price change of A (second‐order effect). We mitigate this concern by restricting the control group to pharmacies whose main competitors are in the control group themselves. This robustness check is informative about whether our choice for the set of main competitors and our definitions of control and treatment groups are adequate.

The maps of the market structure of the OTC market in Lisbon in Appendix S2 show that some retailers exited the market during our study period. Most of these were pharmacies. In robustness checks, we address pharmacy exit in several ways. First, we estimate Equation (1) on a balanced panel of pharmacies. Second, we estimate Equation (1) among pharmacies whose main competitors do not exit the market. 16There can be variation in competition both from entry and from exit of OTC retailers that compose the set of main competitors of a pharmacy (i.e., their three closest neighbors or the retailers within a 400‐m radius). We are interested in variation originating from entry of nonpharmacies, not exit of pharmacies. In order to isolate the former, we focus on a subsample of pharmacies whose main competitors do not exit the market. Therefore, any variation in competition comes from entry of a new retailer.Third, we assess whether experiencing the entry of a nonpharmacy retailer makes pharmacies more likely to exit the market in the future. Specifically, we estimate a logit model whose dependent variable is a binary indicator taking value 1 in case pharmacy *i* exits the market before the next round of data collection and value 0 otherwise. The independent variables are treatment group indicators, year fixed‐effects, and parish fixed‐effects. If the estimates corresponding to the treatment group indicators are not statistically different from zero, then experiencing entry of a supermarket or outlet does not systematically cause pharmacies to exit the market.

### 4.2 Endogeneity of market structure

Our estimates from Equation (1) can only be interpreted as causal if entry and location decisions of nonpharmacies are exogenous. The decision to open a supermarket or outlet in a given location is plausibly unrelated to pharmacy market structure, as OTC drugs are a small subset of their product range. 17In the particular case of supermarket chains, OTC drugs seem to correspond to a small share of total sales. For example, in 2014, the supermarket chain with the largest OTC sales value was Pingo Doce with M8.3 nationwide (Infarmed, 2015). Its total sales value was M3,234 (Jerónimo Martins SGPS SA, 2015). At the time OTC drugs were available at 74 of a total of 380 stores existing Pingo Doce in Portugal. Assuming stores are symmetric, on average, OTC drugs amount to 1.3% of total sales value per store.However, it is more difficult to defend the exogeneity assumption when not all retailers belonging to a given chain apply for a license to sell OTC drugs.

One potential threat is the existence of retailer‐specific unobservables that affect both prices charged by incumbent pharmacies and entry of nonpharmacies. To the extent that these are time invariant, they are captured by the retailer fixed‐effects in our model. However, there can also be *time‐varying*, retailer‐specific unobservables if, for example, certain retailers experience demand shocks due to the natural course of urban development, gentrification of certain neighborhoods, etc. These shocks are difficult to measure at the small geographic level we are working with.

In an attempt to mitigate this concern, we combine propensity score matching with our DID design (Heckman et al. 1997; Smith & Todd, 2005). The underlying intuition is that by matching treated and untreated pharmacies on their propensity score, that is, on their probability of being treated, we make treated and control groups more similar in terms of the observables used in the estimation of the propensity score. Thus, treatment should be random, conditional on those observables. We estimate the propensity score as a function of the levels of competitive pressure and demand faced by each pharmacy prior to experiencing nonpharmacy entry. 18Specifically, demand is measured as population living in the census block where the pharmacy is located, as of 2001. Competitive pressure is measured by the average walking time, in minutes, to the three closest competitors as of 2006 when defining the main competitors of a pharmacy as its three nearest neighbors. When considering all retailers located within a 400‐m radius, competitive pressure is measured by the total number of retailers located inside the 400‐m radius in 2006. Because both measures of demand and competitive pressure are continuous, we categorize them into quintiles and use the categorized variables for the matching. We disregard the groups that were treated already in 2006 in the matching, as for those we do not observe a pretreatment period level of competitive pressure. In Appendix S6, we provide additional technical details on the propensity score matching procedure. We then use the estimated propensity scores to build a matched sample of pharmacies using single neighbor matching. 19As an alternative matching algorithm, we use local linear regression to build the matched sample. The results are shown in Appendix S3.8 and are similar to those for the matched sample using single neighbor matching.Finally, we estimate Equation (1) in this matched sample.

Another potential threat is that, in addition to pharmacies adjusting their prices in the presence of a nonpharmacy, nonpharmacies make location decisions on the basis of prices charged by existing pharmacies in the area. That is, nonpharmacy entrants select where to enter the market on the basis of past prices in the area. For example, entrants might choose to enter in areas where prices are higher as there they could potentially only slightly undercut the incumbents and make higher profits. We address this concern by assuming a sequential game in which in year *t*−1, supermarkets and outlets make joint entry and location decisions for year *t*, taking into account (functions of) *t*−1 prices charged by the pharmacies they would be competing with. Then in year *t*, entry is realized and observed, and all players make their pricing decisions for that year taking entry as given. We have no information on retailers that did not enter the market. Thus, we use the fact that we observe entry in certain locations but not in others. For this analysis, retailers are the relevant unit of observation, and the prices of each of the five OTC drugs are aggregated to generate an OTC bundle price that is retailer‐year specific, $P_{it}=\sum_{k=1}^{5}P_{ikt}$. 20We acknowledge that this is a relatively coarse measure of prices in a geographical area, because we are only considering five OTC drugs, and these five particular OTC drugs might poorly represent the prices of all goods sold by the pharmacies in that area. But this is the best measure we have given the available information.The equation taken to the data is as follows:

(2) $$\begin{array}{ccc} entry_{it}^{*} & =\beta_{0}+\beta_{1}\zeta (P_{i,t-1})+\delta_{t}+\lambda_{j}+\varepsilon_{it}, & \varepsilon_{it}∼iid\;logistic,\\ entry_{it} & =\left\{\begin{array}{ll} 1 & \text{if}\;entry_{it}^{*}>0,\\ 0 & \text{if}\;entry_{it}^{*}\leq 0, \end{array}\right. \end{array}$$

where $entry_{it}^{*}$ is a latent variable representing the probability that pharmacy *i* experiences the entry of a nonpharmacy among its main competitors in year *t*. Although we do not observe this probability, we observe whether a pharmacy experienced nonpharmacy entry at a given point in time, *entry*_*it*_. Thus, *entry*_*it*_ is a binary indicator taking value 1 in case pharmacy *i* experienced the entry of a supermarket or outlet among its main competitors in year *t* and value 0 otherwise. *ζ*(*P*_*t*−1_) is a functional form through which past prices affect entry and location decisions by supermarkets or outlets. *ζ* is, alternatively, the *t*−1 price charged by pharmacy *i* (*P*_*it*−1_), and the ratio between *P*_*it*−1_ and the average *t*−1 price among all retailers operating in Lisbon. The remaining terms are time and parish fixed effects, *δ*_*t*_ and *λ*_*j*_, respectively. *ε*_*it*_ is a logistically distributed error term. Because we take lags of price, the model is estimated using the years 2010 and 2015 only, and the lags are taken with respect to the previous period for which we have data. We estimate separate models for the probability of experiencing entry of a supermarket or an outlet and for our two definitions of main competitors. If the estimates of *β*_1_ are not statistically different from zero in these models, then entry and location decisions of supermarkets and outlets are not driven by past prices charged by pharmacies operating in that location for the five drugs under analysis.

## 5 RESULTS

Table 3 shows our main results. In the first column, we consider the main competitors of a pharmacy to be its three nearest neighbors, and in Column 2, we consider its main competitors to be the retailers located within a 400‐m radius. The results are broadly similar across the two definitions of main competitors. Overall, the entry of a supermarket among the main competitors of a pharmacy is associated with long‐lasting price reductions. In 2010, pharmacies who faced the entry of a supermarket among their main competitors between 2006 and 2010 charged 6%–7% lower prices than those in the control group. In 2015, this very same group of pharmacies was still charging, on average, 4%–6% lower prices than pharmacies in the control group. 21To put these effects into perspective, the entry of a pharmacy chain in India is associated with a 2% price decline among incumbents (Bennett & Yin, 2019), and the entry of Walmart, which charged on average 10% lower prices, is associated with a 1‐1.2% price decrease by its competitors (Basker & Noel, 2009) and a short‐run average city‐level price decrease in the range of 1.5‐3% (Basker, 2005).The effects are insignificant for pharmacies experiencing entry of a supermarket between 2010 and 2015. Although pharmacies experiencing supermarket entry before 2006 charge 2%–3% lower prices than those in the control group both in 2010 and 2015, we do not know how their prices compared with the control group pre‐entry and thus do not put too much emphasis on this result.

**TABLE 3:** Estimates of *θ* from Equation (1), baseline and matched samples

	No matching		Single neighbor matching	
	Three nearest neighbors	400‐m radius	Three nearest neighbors	400‐m radius
	(1)	(2)	(3)	(4)
**DID estimates:**				
2010 × Supermarket entry before 2006 ( $\theta_{1,2010}^{super}$)	−0.027^∗∗∗^			
	(0.008)			
2015 × Supermarket entry before 2006 ( $\theta_{1,2015}^{super}$)	−0.038^∗∗∗^			
	(0.013)			
2010 × Supermarket entry in 2006/10 ( $\theta_{2,2010}^{super}$)	−0.064^∗∗∗^	−0.076^∗∗∗^	−0.055^∗^	−0.053^∗∗^
	(0.019)	(0.015)	(0.033)	(0.026)
2015 × Supermarket entry in 2006/10 ( $\theta_{2,2015}^{super}$)	−0.064^∗∗∗^	−0.038^∗^	−0.080^∗∗^	−0.010
	(0.022)	(0.023)	(0.035)	(0.031)
2015 × Supermarket entry in 2010/15 ( $\theta_{3,2015}^{super}$)	−0.015	−0.025	−0.030	0.009
	(0.016)	(0.017)	(0.027)	(0.025)
2010 × Outlet entry before 2006 ( $\theta_{1,2010}^{outlet}$)	0.013	−0.031		
	(0.020)	(0.022)		
2015 × Outlet entry before 2006 ( $\theta_{1,2015}^{outlet}$)	−0.005	−0.033^∗^		
	(0.010)	(0.019)		
2010 × Outlet entry in 2006/10 ( $\theta_{2,2010}^{outlet}$)	0.009	−0.006	0.019	0.010
	(0.023)	(0.017)	(0.022)	(0.023)
2015 × Outlet entry in 2006/10 ( $\theta_{2,2015}^{outlet}$)	0.015	−0.015	−0.001	−0.024
	(0.021)	(0.020)	(0.025)	(0.026)
2015 × Outlet entry in 2010/15 ( $\theta_{3,2015}^{outlet}$)	−0.001	0.035^∗^	−0.016	0.060^∗∗^
	(0.034)	(0.021)	(0.035)	(0.025)
**Pretreatment trends:**				
2010 × Supermarket entry in 2010/15 ( $\theta_{3,2010}^{super}$)	−0.009	−0.041	0.000	0.020
	(0.027)	(0.033)	(0.024)	(0.024)
2010 × Outlet entry in 2010/15 ( $\theta_{3,2010}^{outlet}$)	−0.007	0.011	0.003	0.040^∗^
	(0.018)	(0.015)	(0.023)	(0.022)
*N*	3,429	3,280	970	960
*R* ^2^	0.912	0.913	0.913	0.903

^*Note*^: Estimates of *θ* ^*super*^ and *θ* ^*outlet*^ based on the estimation of Equation (1) among traditional pharmacies. In Columns 1 and 3, the main competitors of pharmacy *i* are its three nearest neighbors. In Columns 2 and 4, the main competitors of pharmacy *i* are the retailers located with a 400‐m radius. The first two columns estimate the model in the original sample. The last two columns estimate the model on a matched sample of treated and control pharmacies (matching was done using single neighbor matching on propensity scores). We disregard the groups that were treated already in 2006 in the matching, as for those we do not observe a pretreatment period. All specifications include year, drug, and pharmacy fixed‐effects. Standard errors are shown in parenthesis. In Columns 1 and 2, standard errors are clustered at the pharmacy level. In Columns 3 and 4, standard errors are bootstrapped using 30 repetitions drawn cross‐sectionally at the pharmacy level in the original sample.

^Abbreviation^: DID, difference‐in‐differences.

^*^ *p*<0.1.

^**^ *p*<0.05.

^***^ *p*<0.01.

The entry of an outlet among the main competitors of a pharmacy is not associated with price reductions. The finding that incumbent pharmacies react differently to supermarket and outlet entry is consistent with a model in the spirit of Salop (1979), where competition is localized and nonpharmacy entrants can have a cost advantage or cost disadvantage relative to traditional pharmacies. We outline such a model in Appendix S1. In our model, the extent to which pharmacies lower prices after experiencing nonpharmacy entry depends on two distinct forces. On the one hand, there is now a closer competitor that creates downward pressure on incumbent prices. On the other hand, because of the localized nature of competition, incumbents may face a softer or tougher rival at the margin. In case of a more efficient entrant, both these forces go in the direction of lowering pharmacy prices (closer and more efficient rival). In case of a less efficient entrant, the two forces work in opposite directions, and the total impact on pharmacy prices is ambiguous. In our setting, entry by large supermarket chains is likely to be approximated by the low‐cost entrant, reflecting their cost advantage in logistics, management, and, eventually, bargaining power with wholesalers. The entry of outlets, in turn, might be better approximated by the higher‐cost entrant.

In the last two rows of Table 3, we compare the prices charged in 2010 by pharmacies that experience nonpharmacy entry only after 2010 with those charged by pharmacies in the control group. The lack of statistical significance of these estimates supports the plausibility of the common trend assumption, but their magnitude is sometimes not too different from our main effects. Figure 5.5 in Appendix S5 plots raw prices for the two groups of pharmacies treated after 2010 and the control group. These plots do not suggest different trends across groups, though we would need a longer panel to make a stronger claim regarding this matter.

In the last two columns of Table 3, we report the results from estimating Equation (1) on a matched sample of treated and control pharmacies, with matching done using single neighbor matching on propensity scores. The size of the matched sample is considerably smaller than the size of the baseline sample. Although the results obtained with the matched sample go in the same direction as the ones obtained with the baseline sample, some statistical significance is lost.

Table 3.2 in Appendix S3 shows that our results are driven by the most spatially isolated pharmacies as of 2006, who enjoyed some degree of market power before experiencing entry. Our baseline results are robust to estimating the model on a balanced panel of pharmacies, including all retailer types, including pharmacies that are in multiple treatment groups, restricting the sample to pharmacies whose main competitors are in the control group themselves and restricting the sample to pharmacies whose main competitors do not exit the market (Tables 3.5, 3.3, 3.6, 3.4, and 3.7 in Appendix S3, respectively).

We vary the values of *N* and *R* for the definitions of main competitors in Appendix S4. The findings from that exercise convey the fact that competition in the OTC market is very localized. For example, increasing *N* from 3 to 5 shows very few statistically significant price effects following nonpharmacy entry. Similarly, when enlarging the radius within which main competitors are located from 400 to 600 or 800 m, most of the price effects vanish (see Table 4.3 in Appendix S4 for the baseline results and the following tables for robustness checks).

Experiencing the entry of a nonpharmacy retailer does not seem to cause pharmacies to exit the market before the next round of data collection (Table 3.10 in Appendix S3). 22Exit of traditional pharmacies cannot be directly linked to the liberalization of the OTC market, as the share of OTC drugs on total pharmacy revenue is probably too small to produce such an impact. Instead, it is more likely a consequence of the overall economic environment and the squeezing of pharmacy margins on prescription drugs (Barros, 2012). This is consistent with the figures in Table 1, showing that the vast majority of the pharmacies who exited the market were in the control group. In Table 7.1 in Appendix S7, we provide a brief overview of the main regulations affecting pharmacy profitability that were passed between 2005 and 2015.


Finally, Table 4 shows the results of the reduced‐form entry model. These do not support the claim that nonpharmacies make entry decisions on the basis of the prices charged by pharmacies already operating in that area because the estimate of *β*_1_ in Equation (2) is never statistically significant.

**TABLE 4:** Results from the estimation of the reduced‐form entry model

Main competitors	*ζ*(*P*_*t*−1_) specification	Supermarket	Outlet
Three nearest neighbors	*P*_*it*−1_	−0.771	−7.079
		(1.533)	(1.027)
Three nearest neighbors	*P*_*it*−1_ relatively to average market price	−0.999	−1.289
		(4.293)	(1.262)
400‐m radius	*P*_*it*−1_	−0.865	0.665
		(2.226)	(0.836)
400‐m radius	*P*_*it*−1_ relatively to average market price	−1.432	0.548
		(7.868)	(0.820)

^*Note*^: Marginal effects of *β*_1_ from RE logit estimation of Equation (2), with dependent variable being an indicator for facing the entry of a supermarket (Column 1) and an outlet (Column 2). There are two panels. The top panel takes the main competitors of pharmacy *i* as its three nearest neighbors. The bottom panel takes the main competitors of pharmacy *i* as the retailers located within a 400‐m radius. In each of the panels, the first row tests whether pharmacy *i* facing the entry of a supermarket/outlet among its main competitors depends on the prices it charged in the previous period, *ζ*(*P*_*t*−1_)=*P*_*it*−1_. The corresponding figures can be interpreted as the percentage‐point change in the probability of facing entry associated with a 1% higher OTC bundle price in the previous period. The second row tests whether it depends on the lagged prices of pharmacy *i* relative to the average bundle price in the city of Lisbon. The corresponding figures can be interpreted as the percentage‐point change associated with a 1‐unit increase in the independent variable. Recall that our estimation sample differs according to how we define the set of main competitors of pharmacy *i*, so that a different number of observations is used to obtain each estimate shown on the table. Standard errors shown in parenthesis are clustered at the pharmacy level.

^*^ *p*<0.10.

^**^ *p*<0.05.

^***^ *p*<0.01.

Because our reduced‐form entry model has a very specific functional form, we create bar charts of the share of pharmacies in each of the deciles of current and past prices for the bundle of drugs we analyze. We do this separately by year and by type of nonpharmacy entrant. If entry is in any way related to current or past prices, then these plots should convey a nonrandom relationship. In particular, if entry occurred in locations that were potentially more profitable because they had higher prices, then pharmacies in the highest price deciles would experience the largest shares of entry by nonpharmacies. We find no such pattern (Figures 5.1 and 5.3 in Appendix S5). A similar analysis using deciles of resident population instead of price deciles yields again no clear pattern (Figures 5.2 and 5.4 in Appendix S5).

## 6 CONCLUDING REMARKS

We use unique OTC price data at the retailer level for the city of Lisbon to examine the effects of nonpharmacy entry on the prices of incumbent pharmacies. We show that nonpharmacy entry can be successful at fostering competition and lowering prices charged by pharmacies. However, the extent to which this occurs depends crucially on the type nonpharmacy entrant and, particularly, on their ability to exert competitive pressure on incumbent pharmacies. Supermarkets in our sample charge about 20% lower prices than pharmacies, whereas outlets charge 4% lower prices than pharmacies. This means that supermarkets have a greater ability to exert competitive pressure on pharmacies than outlets.

Our baseline results reflect those differences. Although incumbent pharmacies charge 4%–6% lower prices than the control group after experiencing the entry of a supermarket among their main competitors, they do not seem to react to the entry of an outlet. Furthermore, although incumbent pharmacies lower their prices as a response to supermarket entry, they do not lower prices enough so as to match the prices charged by supermarkets. This findings are in line with predictions from a model in the Salop tradition with nonpharmacy entrants differing from incumbents in their marginal cost.

Our results are specific to retailers operating in the municipality of Lisbon and to the set of drugs and time periods we analyze. They might not generalize to other settings. In particular, price reductions may not occur in rural areas, where entry of supermarkets takes place on a smaller scale. Nevertheless, our study contributes to a deeper understanding of how competition takes place in retail pharmaceutical OTC markets.

## CONFLICT OF INTEREST

The authors have no conflicts of interest to declare.

## Supporting information

OTC_MainFile_RR2.tex

OTC_OnlineAppendix_RR2.pdf

OTC_OnlineAppendix_RR2.tex
