# Supplementary material for: Entry and price competition in the over‐the‐counter drug market after deregulation: Evidence from Portugal
Source: Health Econ. 2020 Jun 8;29(8):865–77. doi: 10.1002/hec.4109 (PMC7384133; doi:10.1002/hec.4109)
Supplement: Supplementary file 2 — OTC_OnlineAppendix_RR2.pdf [file HEC-29-865-s002.pdf]

Entry and price competition in the over-the-counter drug market after deregulation: evidence from Portugal

Online Appendix

S1 Theoretical framework

We highlight the economic effects associated with OTC liberalization using a stylized model of entry and competition in the OTC market. We consider a model in the tradition of Salop (1979), with entry of competitors with marginal cost differences relative to incumbents. This reflects the possibility that different types of non-pharmacy entrants may have a cost-advantage or disadvantage relative to community pharmacies. Entry is exogenously given, to focus on the price effects from entry.

The equilibrium price effects of entry into the OTC market will result from extra competition due to more players in the market and from how hard marginal competition has become. In the Salop model, competition is localized, so entry by a low cost rival creates a downward pressure on prices from both a closer rival and a lower marginal cost, more aggressive, competitor. On the other hand, entry by a higher cost competitor brings a balance between a closer rival and a "softer" (higher marginal cost) competitor. The former drives down equilibrium prices while the latter exerts pressure for increasing equilibrium prices.

We consider exogenous entry instead of the free entry equilibrium as in Salop (1979). The existence of entry fixed costs will limit entry in a trivial way and will not add any particular insight. Our interest lies in the price implications of entry of OTC non-pharmacy retailers with different marginal costs, to generate testable implications.

In our setting, entry by large supermarket chains is likely to be approximated by the low-cost entrant, reflecting their cost advantage in logistics, management and, eventually, bargaining power with wholesalers. In areas where supermarkets enter the OTC market, we expect prices to decrease in pharmacies. The entry of other small OTC retailers, outlets, on the other hand may induce a richer set of effects. If they have marginal costs lower than those of pharmacies, but higher than those of supermarkets, the same qualitative effects described for supermarkets apply, though with lower intensity. More interesting is that, in the presence of higher cost entrants, we cannot rule out that equilibrium prices increase. The competition effect works in the direction of lower prices but the strategic interaction effect due to localized competition works in the direction of higher prices whenever the entrant has higher marginal costs. Thus the empirical prediction on the effect of entry of small OTC retailers on equilibrium prices is ambiguous (in the absence of a strong presumption that such outlets have a marginal cost advantage relative to pharmacies).

The model uses the simplest layout to support the above claims.[1] In the pre-entry equilibrium

[1]Importantly, we deviate from the traditional Salop (1979) model in that we do not we do not have fixed costs in our model. We do not explicitly model fixed costs because we want to focus on the price changes after the entry fo non-pharmacy retailers only to obtain testable implications in reduced form equations, and we do not characterize the equilibrium with free entry and relocation of firms within the circle. This modeling option stems from specific features of the OTC market and our setting that may render the free-entry version do the

we consider two pharmacies symmetrically located on the Salop circumference of length one. Density of consumers (patients) is 1 and uniformly distributed along the circumference. Each consumer has a linear cost t of "travelling" to an OTC retailer. A distance x implies a total travel cost of tx. We use x to index a patient location on the circle relative to the nearest left-side OTC retailer. The distance to the nearest right-side retailer is denoted by $d - x$, and the associated travel cost is $t(d - x)$. The value of d is determined by the location of OTC retailers. With n sellers, $d = 1/n$. Consumers of OTC products are assumed to have no insurance coverage (either public or private) for this type of product.[2]

Traditional pharmacies are assumed to be profit maximizing in their decisions regarding the price of OTC products. Pharmacies have a constant marginal cost, c, of selling an OTC product. Supermarkets and outlets have constant marginal cost given by $c + \Delta^S$ and $c + \Delta^O$, respectively. We assume $\Delta^S < 0$, $\Delta^S < \Delta^O$, and Δ^O can be greater or smaller than 0.

To keep the model as tractable as possible without losing any essential element, we assume that entry occurs in pairs (either two supermarkets or two outlets) and that all locations are symmetrically placed on the Salop circumference. These assumptions can be easily relaxed without changing the qualitative nature of the result. Symmetry allows for far more tractable expressions, from which economic intuition can be obtained.

We first characterize the market equilibrium for two symmetrically located community pharmacies. Demand directed to each pharmacy results from patients located both to its left and right-hand sides. A pharmacy located at point i on the Salop circumference faces demand

$$D_i = \left(\frac{1}{2n} - \frac{p_i - p_{i-1}}{2t}\right) + \left(\frac{1}{2n} - \frac{p_i - p_{i+1}}{2t}\right), \quad (1)$$

where $i - 1$ and $i + 1$ denote the locations of rivals. Note that with two pharmacies only, $p_{i-1} = p_{i+1}$, as the other pharmacy is both the left-side and the right-side competitor.

Profit of each pharmacy is

$$\Pi_i = (p_i - c)\left(\frac{1}{n} - \frac{p_i - p_{i-1}}{t}\right). \quad (2)$$

Maximizing each firms' profit with respect to price and solving for the symmetric price equilibrium, we obtain the standard result of $p^* = (t/2) + c$.

model unsuitable. First, relocation is costly and we do not see firms relocating in the data. Second, pharmacy entry is regulated and non-pharmacy entry also requires approval by the regulator (Infarmed). Third, some supermarket entrants will have fixed location as well in the sense that they were already operating before OTC market liberalization and simply added OTC drugs to their product range. Finally, while we do observe pharmacy exit in the data, that is likely driven by developments in the prescription drug market rather than the OTC market. In our theoretical framework, we are only modeling the OTC segment.

[2]Although some OTC products are covered by the National Health Service in Portugal, most are not.

The next step is the characterization of the post-entry equilibrium. We assume that two non-pharmacy OTC retailers enter the market and locate symmetrically on the circle in relation to pharmacies' location. Moreover, pharmacies do not relocate in response to entry.[3] Our assumption of symmetric entrants also implies that entrants have the same marginal cost (different from pharmacies marginal cost).

Demand directed at retailer i now has to accommodate the existence of more competitors, $d = 1/4$. The profit of a retailer located at i is given by

$$\Pi_i = (p_i - c - \Delta)\left(\frac{1}{4} - \frac{p_i - p_{i-1}}{2t} - \frac{p_i - p_{i+1}}{2t}\right) \quad (3)$$

with $\Delta = 0$ for traditional pharmacies.

Pharmacies face a symmetric situation in their decisions and so do supermarkets (or outlets). Thus, we only need to characterize two equilibrium values of prices, one for each type of retailer. Each pharmacy faces competition by two supermarkets/outlets and each supermarket/outlet faces competition of two pharmacies. The resulting equilibrium prices for incumbent pharmacies (I) and non-pharmacy entrants (E) are:

$$p^I = c + \frac{t}{4} + \frac{\Delta^E}{3}, \qquad p^E = c + \frac{t}{4} + \frac{2\Delta^E}{3} \quad (4)$$

From these equilibrium prices it follows that for $\Delta^E < 0$ (more efficient entrants), $p^E < p^I < p^*$. The direct competition effect of more retailers is captured by the difference $(t - t/4)$ when comparing p^I and p^*. The strategic interaction effect from competition is associated with the term Δ^E. With $\Delta^E > 0$ different possibilities exist. Equilibrium price of pharmacies increases if $\Delta^E > 9/4t$ (and pharmacies have lower price than entrants in this case).

These results provide the conceptual background to guide the interpretation of our empirical findings.

[3]Given our assumption of two entrants, the forces for maximum product differentiation and symmetric locations is compatible with the assumption made. Moreover, it is unlikely that pharmacies will relocate geographically as OTC are a relevant but not the main source of their revenues (and relocation may take place in other dimensions relevant to patients other than geographic distance).

S2 OTC Market Structure in Lisbon

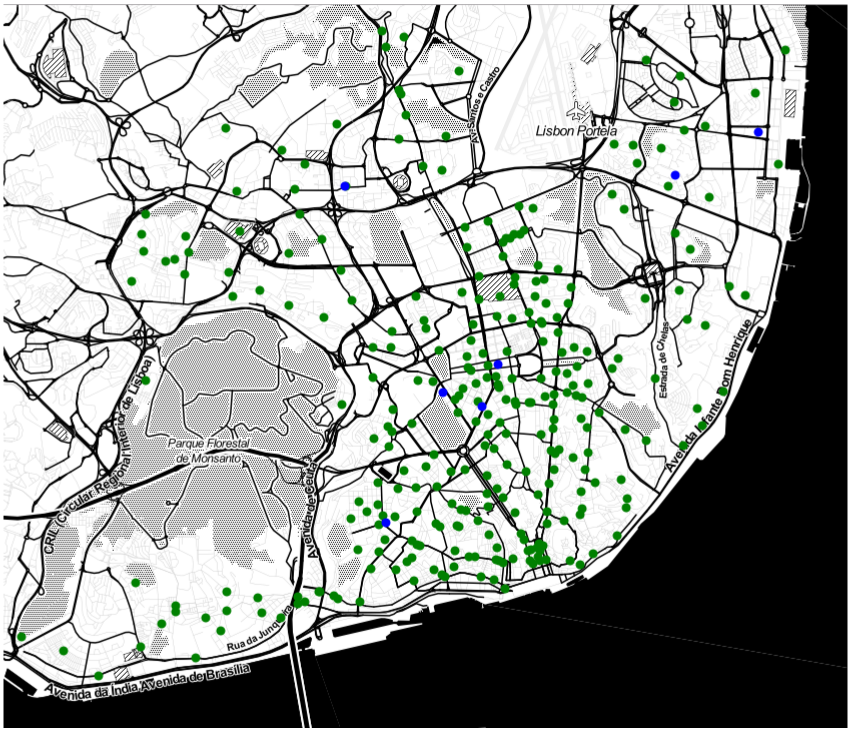

(a) 2006

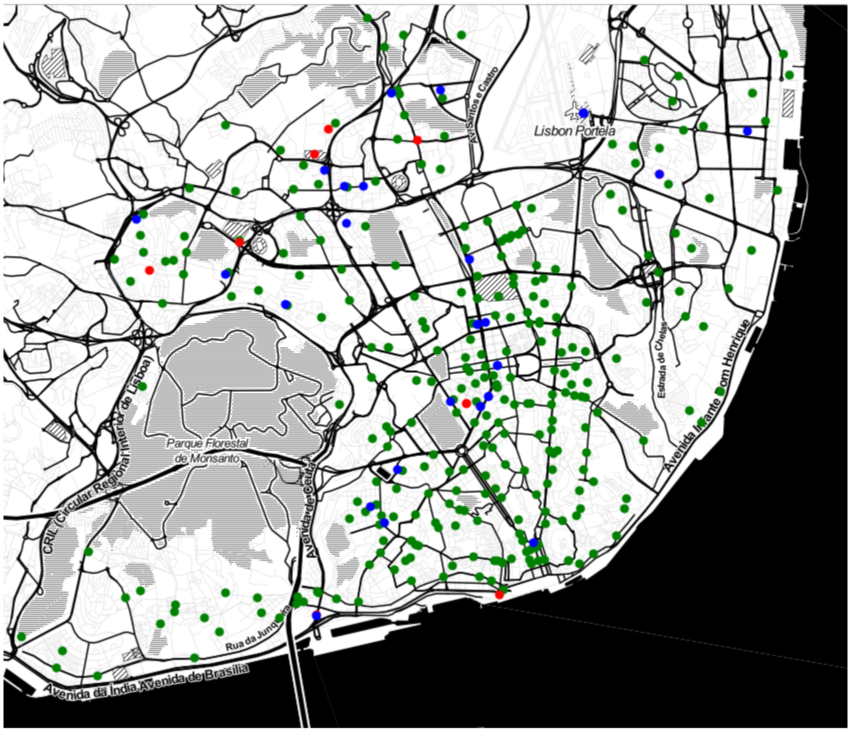

(b) 2010

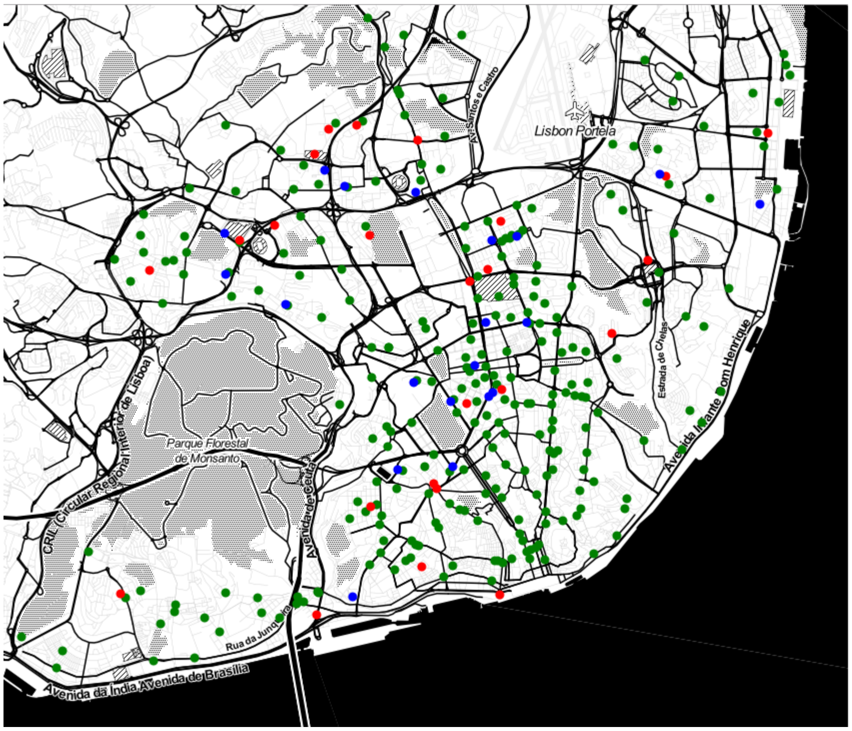

(c) 2015

Figure S2.1: Evolution of OTC market structure in Lisbon

NOTES: Panels (a), (b) and (c) convey the location and type of each OTC retailer active in the Lisbon market as of 2006, 2010, and 2015, respectively. Traditional pharmacies are marked in green, supermarkets are marked in red and outlets are marked in blue. Because some retailers are located very nearby each other, the markers might overlap. In total, there were 301 pharmacies, 1 supermarket, and 8 outlets in 2006; 283 pharmacies, 10 supermarkets, and 25 outlets in 2010; and 259 pharmacies, 25 supermarkets, and 21 outlets in 2015.

S3 Additional tables of results for baseline definitions of main competitors

Remark: The use of a random effects model does not change our basic insights

Table S3.1: Results from estimating equation (1) using random effects

	3 nearest neighbors	400-meter radius
	(1)	(2)
**DID estimates:**		
2010×Supermarket entry before 2006 ($\theta^{super}_{1,2010}$)	-0.026***	
	(0.008)	
2015×Supermarket entry before 2006 ($\theta^{super}_{1,2015}$)	-0.037***	
	(0.013)	
2010×Supermarket entry in 2006/10 ($\theta^{super}_{2,2010}$)	-0.063***	-0.075***
	(0.019)	(0.015)
2015×Supermarket entry in 2006/10 ($\theta^{super}_{2,2015}$)	-0.063***	-0.038
	(0.022)	(0.023)
2015×Supermarket entry in 2010/15 ($\theta^{super}_{3,2015}$)	-0.013	-0.024
	(0.016)	(0.017)
2010×Outlet entry before 2006 ($\theta^{outlet}_{1,2010}$)	0.015	0.023
	(0.020)	(0.022)
2015×Outlet entry before 2006 ($\theta^{outlet}_{1,2015}$)	-0.004	0.022
	(0.009)	(0.020)
2010×Outlet entry in 2006/10 ($\theta^{outlet}_{2,2010}$)	0.011	-0.005
	(0.023)	(0.017)
2015×Outlet entry in 2006/10 ($\theta^{outlet}_{2,2015}$)	0.017	0.014
	(0.021)	(0.019)
2015×Outlet entry in 2010/15 ($\theta^{outlet}_{3,2015}$)	0.001	0.036*
	(0.034)	(0.021)
**Pre-treatment trends:**		
2010×Supermarket entry in 2010/15 ($\theta^{super}_{3,2010}$)	-0.008	-0.039
	(0.027)	(0.033)
2010×Outlet entry in 2010/15 ($\theta^{outlet}_{3,2010}$)	-0.005	0.012
	(0.018)	(0.014)
Observations	3,429	3,280
R^2	0.912	0.913

NOTES: Estimates of θ based on the estimation of equation (1) among pharmacies using random effects. Columns 1 takes the main competitors of pharmacy i as its 3 nearest neighbors and column 2 considers all retailers within a 400-meter radius as main competitors. All models include year, drug, parish, and treatment group fixed-effects. Standard errors shown in parenthesis are clustered at the pharmacy level. $*p < 0.10, **p < 0.05, ***p < 0.01$.

Remark: Our results are driven by the most spatially isolated pharmacies

Table S3.2: Results from estimating equation (1) among the most and least spatially isolated pharmacies in 2006

	Most spatially isolated		Least spatially isolated	
	3 nearest neighbors	400-meter radius	3 nearest neighbors	400-meter radius
	(1)	(2)	(3)	(4)
**DID estimates:**				
2010×Supermarket entry before 2006 ($\theta^{super}_{1,2010}$)	-0.026**			
	(0.010)			
2015×Supermarket entry before 2006 ($\theta^{super}_{1,2015}$)	-0.033**			
	(0.015)			
2010×Supermarket entry in 2006/10 ($\theta^{super}_{2,2010}$)	-0.071***	-0.099***	-0.058*	-0.030***
	(0.022)	(0.018)	(0.030)	(0.010)
2015×Supermarket entry in 2006/10 ($\theta^{super}_{2,2015}$)	-0.078***	-0.074***	-0.036*	-0.013
	(0.018)	(0.024)	(0.019)	(0.009)
2015×Supermarket entry in 2010/15 ($\theta^{super}_{3,2015}$)	-0.033**	-0.047	-0.007	-0.010
	(0.016)	(0.036)	(0.025)	(0.019)
2010×Outlet entry before 2006 ($\theta^{outlet}_{1,2010}$)	0.008	-0.026	0.013	0.076***
	(0.025)	(0.021)	(0.021)	(0.019)
2015×Outlet entry before 2006 ($\theta^{outlet}_{1,2015}$)	-0.003	-0.015	-0.010	0.067***
	(0.013)	(0.015)	(0.010)	(0.021)
2010×Outlet entry in 2006/10 ($\theta^{outlet}_{2,2010}$)	-0.014	-0.054***	0.030	0.032
	(0.026)	(0.017)	(0.030)	(0.021)
2015×Outlet entry in 2006/10 ($\theta^{outlet}_{2,2015}$)	0.006	-0.018	0.003	0.045**
	(0.025)	(0.031)	(0.033)	(0.021)
2015×Outlet entry in 2010/15 ($\theta^{outlet}_{3,2015}$)	0.067	0.076*	-0.005	0.026
	(0.052)	(0.039)	(0.034)	(0.023)
**Pre-treatment trends:**				
2010×Supermarket entry in 2010/15 ($\theta^{super}_{3,2010}$)	-0.041	-0.008	0.017	-0.053
	(0.032)	(0.020)	(0.025)	(0.046)
2010×Outlet entry in 2010/15 ($\theta^{outlet}_{3,2010}$)	-0.016	-0.005	-0.007	0.020
	(0.037)	(0.039)	(0.019)	(0.015)
Observations	1,257	924	1,752	1,287
R^2	0.921	0.922	0.918	0.919

NOTES: Estimates of θ based on the estimation of equation (1) among pharmacies located in areas where market structure is the most and the lest concentrated. Columns 1 and 3 take the main competitors of pharmacy i as its 3 nearest neighbors and columns 2 and 4 consider all retailers within a 400-meter radius as main competitors. In columns 1 and 3 the sample was restricted to pharmacies whose walking time (in minutes) to their 3rd nearest competitor is above and the sample median in 2006, respectively. In columns 2 and 4 the sample was restricted to pharmacies whose number of competitors within a 400-meter radius in 2006 is below and above the sample median, respectively. All models include year, drug, and pharmacy fixed-effects. Standard errors shown in parenthesis are clustered at the pharmacy level. $*p < 0.10, **p < 0.05, ***p < 0.01$.

Remark: Our results are robust to including all retailer types in the estimation

Table S3.3: Results from estimating equation (1) among all retailer types

	3 nearest neighbors	400-meter radius
	(1)	(2)
**DID estimates:**		
2010×Supermarket entry before 2006 ($\theta^{super}_{1,2010}$)	-0.029***	
	(0.008)	
2015×Supermarket entry before 2006 ($\theta^{super}_{1,2015}$)	-0.034**	
	(0.013)	
2010×Supermarket entry in 2006/10 ($\theta^{super}_{2,2010}$)	-0.066***	-0.076***
	(0.019)	(0.015)
2015×Supermarket entry in 2006/10 ($\theta^{super}_{2,2015}$)	-0.060***	-0.038*
	(0.022)	(0.023)
2015×Supermarket entry in 2010/15 ($\theta^{super}_{3,2015}$)	-0.011	-0.025
	(0.016)	(0.017)
2010×Outlet entry before 2006 ($\theta^{outlet}_{1,2010}$)	-0.008	0.031
	(0.027)	(0.022)
2015×Outlet entry before 2006 ($\theta^{outlet}_{1,2015}$)	0.000	0.033*
	(0.019)	(0.019)
2010×Outlet entry in 2006/10 ($\theta^{outlet}_{2,2010}$)	0.008	-0.006
	(0.023)	(0.017)
2015×Outlet entry in 2006/10 ($\theta^{outlet}_{2,2015}$)	0.019	0.015
	(0.021)	(0.020)
2015×Outlet entry in 2010/15 ($\theta^{outlet}_{3,2015}$)	0.003	0.035*
	(0.034)	(0.021)
**Pre-treatment trends:**		
2010×Supermarket entry in 2010/15 ($\theta^{super}_{3,2010}$)	-0.011	-0.040
	(0.027)	(0.033)
2010×Outlet entry in 2010/15 ($\theta^{outlet}_{3,2010}$)	-0.008	0.011
	(0.018)	(0.015)
Observations	3,851	3,280
R^2	0.905	0.913

NOTES: Estimates of θ based on the estimation of equation (1) among all retailer types: traditional pharmacies, supermarkets and outlets. Column 1 takes the main competitors of retailer i as its 3 nearest neighbors. Column 2 considers as main competitors of retailer i all retailers located within a 400-meter radius. All models include year, drug, and retailer fixed-effects. Standard errors shown in parenthesis are clustered at the retailer level. $*p < 0.10, **p < 0.05, ***p < 0.01$.

Remark: Our results are robust to restricting the estimation to pharmacies whose competitors are all in the control group

Table S3.4: Results from estimating equation (1) among pharmacies whose competitors are all in the control group

	3 nearest neighbors	400-meter radius
	(1)	(2)
**DID estimates:**		
2010×Supermarket entry before 2006 ($\theta^{super}_{1,2010}$)	-0.026***	
	(0.009)	
2015×Supermarket entry before 2006 ($\theta^{super}_{1,2015}$)	-0.038***	
	(0.014)	
2010×Supermarket entry in 2006/10 ($\theta^{super}_{2,2010}$)	-0.049**	-0.073***
	(0.020)	(0.015)
2015×Supermarket entry in 2006/10 ($\theta^{super}_{2,2015}$)	-0.058***	-0.037
	(0.020)	(0.023)
2015×Supermarket entry in 2010/15 ($\theta^{super}_{3,2015}$)	-0.015	-0.024
	(0.017)	(0.018)
2010×Outlet entry before 2006 ($\theta^{outlet}_{1,2010}$)	-0.006	0.034
	(0.025)	(0.022)
2015×Outlet entry before 2006 ($\theta^{outlet}_{1,2015}$)	0.000	0.034*
	(0.022)	(0.019)
2010×Outlet entry in 2006/10 ($\theta^{outlet}_{2,2010}$)	0.011	-0.003
	(0.023)	(0.017)
2015×Outlet entry in 2006/10 ($\theta^{outlet}_{2,2015}$)	0.015	0.017
	(0.021)	(0.020)
2015×Outlet entry in 2010/15 ($\theta^{outlet}_{3,2015}$)	-0.001	0.0.37*
	(0.028)	(0.021)
**Pre-treatment trends:**		
2010×Supermarket entry in 2010/15 ($\theta^{super}_{3,2010}$)	-0.022	0.014
	(0.023)	(0.015)
2010×Outlet entry in 2010/15 ($\theta^{outlet}_{3,2010}$)	-0.000	0.010
	(0.017)	(0.015)
Observations	2,455	2,764
R^2	0.915	0.915

NOTES: Estimates of θ based on the estimation of equation (1) among pharmacies whose competitors are all in the control group. Column 1 takes the main competitors of pharmacy i as its 3 nearest neighbors. Column 2 considers as main competitors of pharmacy i all retailers located within a 400-meter radius. All models include year, drug, and pharmacy fixed-effects. Standard errors shown in parenthesis are clustered at the pharmacy level. $*p < 0.10, **p < 0.05, ***p < 0.01$.

Remark: Our results are robust to restricting the estimation to a balanced panel of pharmacies

Table S3.5: Results from estimating equation (1) in a balanced panel of pharmacies

	3 nearest neighbors	400-meter radius
	(1)	(2)
**DID estimates:**		
2010×Supermarket entry before 2006 ($\theta^{super}_{1,2010}$)	-0.022***	
	(0.008)	
2015×Supermarket entry before 2006 ($\theta^{super}_{1,2015}$)	-0.037***	
	(0.013)	
2010×Supermarket entry in 2006/10 ($\theta^{super}_{2,2010}$)	-0.046**	-0.071***
	(0.020)	(0.015)
2015×Supermarket entry in 2006/10 ($\theta^{super}_{2,2015}$)	-0.057***	-0.035
	(0.020)	(0.023)
2015×Supermarket entry in 2010/15 ($\theta^{super}_{3,2015}$)	-0.011	-0.021
	(0.017)	(0.019)
2010×Outlet entry before 2006 ($\theta^{outlet}_{1,2010}$)	-0.005	0.039
	(0.025)	(0.025)
2015×Outlet entry before 2006 ($\theta^{outlet}_{1,2015}$)	0.000	0.038*
	(0.021)	(0.021)
2010×Outlet entry in 2006/10 ($\theta^{outlet}_{2,2010}$)	0.014	-0.011
	(0.023)	(0.018)
2015×Outlet entry in 2006/10 ($\theta^{outlet}_{2,2015}$)	0.016	0.014
	(0.021)	(0.020)
2015×Outlet entry in 2010/15 ($\theta^{outlet}_{3,2015}$)	-0.000	0.038*
	(0.028)	(0.021)
**Pre-treatment trends:**		
2010×Supermarket entry in 2010/15 ($\theta^{super}_{3,2010}$)	-0.016	-0.037
	(0.023)	(0.034)
2010×Outlet entry in 2010/15 ($\theta^{outlet}_{3,2010}$)	0.002	0.015
	(0.017)	(0.015)
Observations	2,265	2,043
R^2	0.923	0.923

NOTES: Estimates of θ based on the estimation of equation (1) among pharmacies who are observed at all time periods (2006, 2010, and 2015). Column 1 takes the main competitors of pharmacy i as its 3 nearest neighbors. Column 2 considers as main competitors of pharmacy i all retailers located within a 400-meter radius. All models include year, drug, and pharmacy fixed-effects. Standard errors shown in parenthesis are clustered at the pharmacy level. $*p < 0.10, **p < 0.05, ***p < 0.01$.

Remark: Our results are robust to including pharmacies experiencing multiple treatments in the estimation

Table S3.6: Results from estimating equation (1) with non-mutually exclusive treatments

	3 nearest neighbors (1)	400-meter radius (2)
**DID estimates:**		
2010×Supermarket entry before 2006 ($\theta^{super}_{1,2010}$)	-0.027***	
	(0.008)	
2015×Supermarket entry before 2006 ($\theta^{super}_{1,2015}$)	-0.038***	
	(0.013)	
2010×Supermarket entry in 2006/10 ($\theta^{super}_{2,2010}$)	-0.064***	-0.077***
	(0.019)	(0.015)
2015×Supermarket entry in 2006/10 ($\theta^{super}_{2,2015}$)	-0.064***	-0.040*
	(0.022)	(0.023)
2015×Supermarket entry in 2010/15 ($\theta^{super}_{3,2015}$)	-0.018	-0.061***
	(0.015)	(0.021)
2010×Outlet entry before 2006 ($\theta^{outlet}_{1,2010}$)	0.013	0.030
	(0.020)	(0.022)
2015×Outlet entry before 2006 ($\theta^{outlet}_{1,2015}$)	-0.005	0.031
	(0.010)	(0.019)
2010×Outlet entry in 2006/10 ($\theta^{outlet}_{2,2010}$)	0.009	-0.007
	(0.023)	(0.017)
2015×Outlet entry in 2006/10 ($\theta^{outlet}_{2,2015}$)	0.015	0.014
	(0.021)	(0.020)
2015×Outlet entry in 2010/15 ($\theta^{outlet}_{3,2015}$)	-0.004	0.017
	(0.031)	(0.019)
**Pre-treatment trends:**		
2010×Supermarket entry in 2010/15 ($\theta^{super}_{3,2010}$)	-0.012	-0.053**
	(0.024)	(0.021)
2010×Outlet entry in 2010/15 ($\theta^{outlet}_{3,2010}$)	-0.010	0.004
	(0.017)	(0.014)
Observations	3,769	3,679
R^2	0.909	0.909

NOTES: Estimates of θ based on the estimation of equation (1) among pharmacies, without imposing mutually exclusivity of treatment groups. Column 1 takes the main competitors of pharmacy i as its 3 nearest neighbors. Column 2 considers as main competitors of pharmacy i all retailers located within a 400-meter radius. All models include year, drug, and pharmacy fixed-effects as well as interactions between different treatment groups. Standard errors shown in parenthesis are clustered at the pharmacy level. $*p < 0.10, **p < 0.05, ***p < 0.01$.

Remark: Our results are robust to restricting the estimation to pharmacies whose main competitors do not exit the market

Table S3.7: Results from estimating equation (1) among pharmacies whose main competitors do not exit

	3 nearest neighbors	400-meter radius
	(1)	(2)
**DID estimates:**		
2010×Supermarket entry before 2006 ($\theta^{super}_{1,2010}$)	-0.023**	
	(0.009)	
2015×Supermarket entry before 2006 ($\theta^{super}_{1,2015}$)	-0.039***	
	(0.014)	
2010×Supermarket entry in 2006/10 ($\theta^{super}_{2,2010}$)	-0.125***	-0.091***
	(0.007)	(0.015)
2015×Supermarket entry in 2006/10 ($\theta^{super}_{2,2015}$)	-0.056***	-0.046
	(0.008)	(0.028)
2015×Supermarket entry in 2010/15 ($\theta^{super}_{3,2015}$)	-0.031**	0.007
	(0.015)	(0.021)
2010×Outlet entry before 2006 ($\theta^{outlet}_{1,2010}$)	0.055***	0.042**
	(0.007)	(0.018)
2015×Outlet entry before 2006 ($\theta^{outlet}_{1,2015}$)	-0.013*	0.037*
	(0.008)	(0.021)
2010×Outlet entry in 2006/10 ($\theta^{outlet}_{2,2010}$)	-0.020	-0.011
	(0.036)	(0.018)
2015×Outlet entry in 2006/10 ($\theta^{outlet}_{2,2015}$)	0.013	0.011
	(0.029)	(0.021)
2015×Outlet entry in 2010/15 ($\theta^{outlet}_{3,2015}$)	-0.059	0.024
	(0.046)	(0.021)
**Pre-treatment trends:**		
2010×Supermarket entry in 2010/15 ($\theta^{super}_{3,2010}$)	-0.068	-0.027
	(0.050)	(0.030)
2010×Outlet entry in 2010/15 ($\theta^{outlet}_{3,2010}$)	-0.012	0.002
	(0.016)	(0.016)
Observations	1,975	2,631
R^2	0.912	0.914

NOTES: Estimates of θ based on the estimation of equation (1) among pharmacies whose main competitors do not exit the market during the time horizon under analysis. Column 1 takes the main competitors of pharmacy i as its 3 nearest neighbors. Column 2 considers as main competitors of pharmacy i all retailers located within a 400-meter radius. All models include year, drug, and pharmacy fixed-effects. Standard errors shown in parenthesis are clustered at the pharmacy level. $*p < 0.10, **p < 0.05, ***p < 0.01$.

Remark: Statistical significance is often lost when estimating our model in a PS-matched sample using local linear regression

Table S3.8: Results from estimating equation (1) in a PS-matched sample using local linear regression

	3 nearest neighbors	400-meter radius
	(1)	(2)
**DID estimates:**		
2010×Supermarket entry in 2006/10 ($\theta^{super}_{2,2010}$)	-0.055	-0.053**
	(0.056)	(0.026)
2015×Supermarket entry in 2006/10 ($\theta^{super}_{2,2015}$)	-0.080*	-0.010
	(0.048)	(0.031)
2015×Supermarket entry in 2010/15 ($\theta^{super}_{3,2015}$)	-0.030	0.008
	(0.044)	(0.030)
2010×Outlet entry in 2006/10 ($\theta^{outlet}_{2,2010}$)	0.019	0.011
	(0.028)	(0.024)
2015×Outlet entry in 2006/10 ($\theta^{outlet}_{2,2015}$)	-0.001	0.024
	(0.050)	(0.027)
2015×Outlet entry in 2010/15 ($\theta^{outlet}_{3,2015}$)	-0.016	0.060**
	(0.066)	(0.024)
**Pre-treatment trends:**		
2010×Supermarket entry in 2010/15 ($\theta^{super}_{3,2010}$)	0.000	0.021
	(0.045)	(0.025)
2010×Outlet entry in 2010/15 ($\theta^{outlet}_{3,2010}$)	0.002	0.040*
	(0.065)	(0.021)
Observations	970	1,180
R^2	0.913	0.904

NOTES: Estimates of θ based on the estimation of equation (1) in a matched sample of pharmacies in the treated groups and pharmacies in the control group. Matching was done on propensity scores using local linear regression. Column 1 takes the main competitors of pharmacy i as its 3 nearest neighbors. Column 2 considers as main competitors of pharmacy i all retailers located within a 400-meter radius. All models include year, drug, and pharmacy fixed-effects. Standard errors shown in parenthesis are bootstrapped using 30 repetitions, drawn cross-sectionally at the pharmacy level in the original sample. $*p < 0.10, **p < 0.05, ***p < 0.01$.

Remark: The statistical significance of ou results is robust to using two-way clustering by drug and pharmacy

Table S3.9: Results from estimating equation (1) with 2-way clustering of standard errors

	3 nearest neighbors	400m radius
	(1)	(2)
**DiD estimates:**		
2010×Supermarket entry before 2006 ($\theta^{super}_{1,2010}$)	-0.027	
	(0.023)	
2015×Supermarket entry before 2006 ($\theta^{super}_{1,2015}$)	-0.038**	
	(0.015)	
2010×Supermarket entry in 2006/10 ($\theta^{super}_{2,2010}$)	-0.064***	-0.076**
	(0.012)	(0.019)
2015×Supermarket entry in 2006/10 ($\theta^{super}_{2,2015}$)	-0.064***	-0.038**
	(0.012)	(0.013)
2015×Supermarket entry in 2010/15 ($\theta^{super}_{3,2015}$)	-0.015	-0.025
	(0.010)	(0.013)
2010×Outlet entry before 2006 ($\theta^{outlet}_{1,2010}$)	0.013	0.031
	(0.009)	(0.018)
2015×Outlet entry before 2006 ($\theta^{outlet}_{1,2015}$)	-0.005	0.033**
	(0.005)	(0.011)
2010×Outlet entry in 2006/10 ($\theta^{outlet}_{2,2010}$)	0.009	-0.006
	(0.022)	(0.013)
2015×Outlet entry in 2006/10 ($\theta^{outlet}_{2,2015}$)	0.015	0.015
	(0.015)	(0.016)
2015×Outlet entry in 2010/15 ($\theta^{outlet}_{3,2015}$)	-0.001	0.035
	(0.030)	(0.019)
**Pre-treatment trends:**		
2010×Supermarket entry in 2010/15 ($\theta^{super}_{3,2010}$)	-0.009	-0.040
	(0.024)	(0.031)
2010×Outlet entry in 2010/15 ($\theta^{outlet}_{3,2010}$)	-0.007	0.011
	(0.021)	(0.016)
Observations	3,429	3,280
R^2	0.912	0.919

NOTES: Estimates of θ based on the estimation of equation (1) among traditional pharmacies. In column 1 the main competitors of pharmacy i are its 3 nearest neighbors. In column 2 the main competitors of pharmacy i are the retailers located with a 400-meter radius. All specifications include year, drug, and pharmacy fixed-effects. Standard errors are shown in parenthesis are clustered at the pharmacy and drug level. * $p < 0.1$, ** $p < 0.05$, *** $p < 0.01$

Remark: We find no evidence that experiencing non-pharmacy entry makes pharmacies more likely to exit the market before the next data collection round

Table S3.10: Does experiencing non-pharmacy entry make pharmacies more likely to exit next period?

	Number of nearest neighbors (N)		Radius (R)		
	4	5	400m	600m	800m
	(1)	(2)	(3)	(4)	(5)
Supermarket entry before 2006					
Supermarket entry in 2006/10				-0.132	-0.033
				(0.0123)	(0.101)
Outlet entry before 2006	-0.137	0.071	0.006	0.095	0.042
	(0.135)	(0.130)	(0.099)	(0.073)	(0.096)
Outlet entry in 2006/10	-0.143	-0.109	-0.128	-0.121	-0.145*
	(0.111)	(0.091)	(0.086)	(0.085)	(0.086)
Observations	380	356	368	328	265

NOTES: Marginal effects from a logit regression of a binary variable equaling 1 for pharmacies that exited the market before the next round of data collection and 0 otherwise, on treatment group indicators, parish fixed-effects, and year fixed-effects. Columns 1 and 2 take the main competitors of pharmacy i as its N nearest neighbors, with N=4 and N=5, respectively. For N=1,2,3 there is not enough variation to estimate the model because none of pharmacies experiencing non-pharmacy entry among 1,2, and 3 nearest competitors exits the market. Columns 3, 4, and 5 take all retailers located within a 400, 600, and 800-meter radius as main competitors of pharmacy i. Regardless of the definition of main competitors used, no pharmacies experiencing supermarket entry among their main competitors before 2006 exited the market so the corresponding coefficients cannot be estimated. Similarly, when using $N = 4$, $N = 5$, and $R = 400$, none of the pharmacies that experienced entry of a supermarket among their main competitors between 2006 and 2010 exited the market so these coefficients cannot be estimated either. All models include year and parish fixed-effects. Standard errors shown in parenthesis are clustered at the pharmacy level. $*p < 0.10, **p < 0.05, ***p < 0.01$.

S4 Results for other definitions of main competitors

Remark: Enlarging the set of main competitors of a pharmacy (by including a larger number of nearest neighbors or increasing the radius distance) yields few statistically significant price effects, suggesting competition in the OTC market is fairly localized.

Table S4.1: Sample composition for alternative definitions of main competitors

Main competitors	Group	2006	2010	2015
	Control Group	190	258	234
	Supermarket entry before 2006	0	0	0
Nearest neighbor	Supermarket entry in 2006/10	4	4	4
	Supermarket entry in 2010/15	4	4	4
	Outlet entry before 2006	8	8	8
	Outlet entry in 2006/10	10	10	10
	Outlet entry in 2010/15	15	15	15
	Total	231	299	275
	Control Group	167	235	212
	Supermarket entry before 2006	1	1	1
2 nearest neighbors	Supermarket entry in 2006/10	3	3	3
	Supermarket entry in 2010/15	8	8	8
	Outlet entry before 2006	9	9	9
	Outlet entry in 2006/10	8	8	8
	Outlet entry in 2010/15	9	9	9
	Total pharmacies	205	273	250
	Control Group	138	207	185
	Supermarket entry before 2006	3	3	3
4 nearest neighbors	Supermarket entry in 2006/10	5	5	5
	Supermarket entry in 2010/15	19	19	19
	Outlet entry before 2006	13	12	12
	Outlet entry in 2006/10	10	10	9
	Outlet entry in 2010/15	18	18	18
	Total pharmacies	206	274	251

Continued on next page

Table S4.1 – *Continued from previous page*

Main competitors	Group	2006	2010	2015
	Control Group	109	179	159
	Supermarket entry before 2006	0	0	0
5 nearest neighbors	Supermarket entry in 2006/10	5	5	5
	Supermarket entry in 2010/15	18	18	18
	Outlet entry before 2006	10	8	7
	Outlet entry in 2006/10	14	14	12
	Outlet entry in 2010/15	18	18	18
	Total pharmacies	174	242	219
	Control Group	94	166	150
	Supermarket entry before 2006	1	1	1
600m radius	Supermarket entry in 2006/10	9	9	8
	Supermarket entry in 2010/15	12	12	12
	Outlet entry before 2006	13	9	7
	Outlet entry in 2006/10	17	17	14
	Outlet entry in 2010/15	12	12	12
	Total pharmacies	158	226	204
	Control Group	68	141	129
	Supermarket entry before 2006	0	0	0
800m radius	Supermarket entry in 2006/10	9	9	7
	Supermarket entry in 2010/15	8	8	8
	Outlet entry before 2006	12	7	6
	Outlet entry in 2006/10	21	21	18
	Outlet entry in 2010/15	10	10	10
	Total pharmacies	128	196	178

NOTES: The table shows the number of pharmacies included in the estimation sample, for alternative definitions of main competitors of a pharmacy: the N nearest neighbors with N=1,2,4,5 in the top four panels, and the retailers located within a radius R of 600 and 800 meters in the two bottom panels. The lower number of pharmacies in the control group in 2006 is a consequence of missing price data for that year, as discussed in Section 3. In addition, the number of pharmacies used in the estimation sample changes with the definition of main competitors because we are focusing on samples of pharmacies for which each treatment is mutually exclusive. Thus, a longer radius (or more nearest neighbors) means higher chances that a pharmacy falls into more than one treatment group and is excluded from the analysis.

Table S4.2: Testing for differences at baseline for alternative definitions of main competitors

Variable	Control	Eventually Treated	Difference	P-value
Main competitors: nearest neighbor				
Price *Aspirina 500mg* (€)	3.041	2.873	0.167*	0.018
Price *Cêgripe* (€)	4.292	4.273	0.019	0.745
Price *Trifene200* (€)	3.326	3.271	0.055	0.428
Price *Mebocaína Forte* (€)	4.664	4.582	0.082	0.295
Price *Tantum Verde* (€)	4.970	4.864	0.107	0.309
Avg distance to nearest neighbor (km)	0.309	0.434	-0.125*	0.059
Avg time to nearest neighbor (min)	3.729	5.077	-1.348	0.120
Population in census block (as of 2001)	609.516	698.308	-88.792	0.124
Main competitors: 2 nearest neighbors				
Price *Aspirina 500mg* (€)	3.030	3.045	-0.015	0.772
Price *Cêgripe* (€)	4.302	4.273	0.019	0.745
Price *Trifene200* (€)	3.336	3.268	0.069	0.167
Price *Mebocaína Forte* (€)	4.663	4.635	0.028*	0.062
Price *Tantum Verde* (€)	4.972	4.899	0.073	0.037
Avg distance to 2 nearest neighbors (km)	0.154	0.188	-0.034	0.142
Avg time to 2 nearest neighbors (min)	4.406	5.357	-0.951	0.128
Population in census block (as of 2001)	598.024	723.286	-125.262***	0.002
Main competitors: 4 nearest neighbors				
Price *Aspirina 500mg* (€)	3.036	2.997	0.040	0.321
Price *Cêgripe* (€)	4.314	4.224	0.089**	0.013
Price *Trifene200* (€)	3.415	3.307	0.035	0.411
Price *Mebocaína Forte* (€)	4.671	4.667	0.004	0.931
Price *Tantum Verde* (€)	4.979	4.918	0.061	0.356
Avg distance to 4 nearest neighbors (km)	0.311	0.323	-0.012	0.717
Avg time to 4 nearest neighbors (min)	5.796	5.860	-0.064	0.920
Population in census block (as of 2001)	591.058	662.233	-71.175**	0.040

Continued on next page

Table S4.2 – *Continued from previous page*

Variable	Control	Eventually Treated	Difference	P-value
Main competitors: 5 nearest neighbors				
Price *Aspirina 500mg* (€)	3.033	3.013	0.020	0.588
Price *Cêgripe* (€)	4.325	4.260	0.065*	0.063
Price *Trifene200* (€)	3.340	3.326	0.014	0.732
Price *Mebocaína Forte* (€)	4.681	4.697	-0.015	0.725
Price *Tantum Verde* (€)	4.993	4.940	0.053	0.418
Avg distance to 5 nearest neighbors (km)	0.510	0.512	-0.002	0.966
Avg time to 5 nearest neighbors (min)	6.385	6.249	0.136	0.836
Population in census block (as of 2001)	588.156	618.763	-30.608	0.340
Main competitors: 600-meter radius				
Price *Aspirina 500mg* (€)	3.022	3.019	0.002	0.958
Price *Cêgripe* (€)	4.321	4.238	0.084*	0.052
Price *Trifene200* (€)	3.337	3.299	0.039	0.382
Price *Mebocaína Forte* (€)	4.666	4.678	-0.012	0.811
Price *Tantum Verde* (€)	4.987	4.915	0.072	0.308
Number of retailers within radius	10.376	7.108	3.268***	0.002
Population in Census section (as of 2001)	594.101	651.243	-57.142	0.142
Main competitors: 800-meter radius				
Price *Aspirina 500mg* (€)	3.008	3.027	0.019	0.688
Price *Cêgripe* (€)	4.310	4.253	0.057	0.224
Price *Trifene200* (€)	3.324	3.306	0.018	0.697
Price *Mebocaína Forte* (€)	4.657	4.673	-0.016	0.761
Price *Tantum Verde* (€)	4.977	4.884	0.093	0.244
Number of retailers within radius	14.153	10.448	3.705**	0.010
Population in Census section (as of 2001)	588.329	653.103	-64.774	0.151

NOTES: The table conveys the mean of several variables of interest in 2006 for several alternative definitions of main competitors. In the top four panels, the main competitors of a pharmacy are its N nearest neighbors, with N=1,2,4,5, respectively. In the two bottom panels, the main competitors of a pharmacy are all retailers located inside a 600 and 800-meter radius, respectively. For each panel, the first column reports averages across pharmacies belonging to the control group. The second column reports averages across pharmacies which were not yet treated in 2006, but will eventually face the entry of a non-pharmacy amongst their main competitors, thus grouping together pharmacies facing the entry of a supermarket or an outlet either between 2006 and 2010, or between 2010 and 2015. Pharmacies already treated in 2006 is not accounted for in this table because they are not observed prior to treatment. Column 3 computes the difference of columns 1 and 2, and column 4 shows the corresponding two-sided p-value.

Table S4.3: Results from estimating equation (1) with alternative definitions of main competitors

	Number of nearest neighbors				Radius	
	1	2	4	5	600m	800m
	(1)	(2)	(3)	(4)	(5)	(6)
**DID estimates:**						
2010×Supermarket entry before 2006 ($\theta^{super}_{1,2010}$)		-0.038***	-0.037***		-0.036***	
		(0.005)	(0.006)		(0.007)	
2015×Supermarket entry before 2006 ($\theta^{super}_{1,2015}$)		-0.023***	-0.023***		-0.020***	
		(0.005)	(0.006)		(0.008)	
2010×Supermarket entry in 2006/10 ($\theta^{super}_{2,2010}$)	-0.063**	-0.094***	-0.082***	-0.049	-0.022	-0.037
	(0.027)	(0.016)	(0.023)	(0.031)	(0.026)	(0.032)
2015×Supermarket entry in 2006/10 ($\theta^{super}_{2,2015}$)	-0.062**	-0.095***	-0.050*	-0.044*	-0.038*	-0.030
	(0.028)	(0.017)	(0.028)	(0.032)	(0.020)	(0.023)
2015×Supermarket entry in 2010/15 ($\theta^{super}_{3,2015}$)	-0.035*	-0.037***	0.029	-0.028**	-0.010	-0.045*
	(0.020)	(0.012)	(0.018)	(0.015)	(0.021)	(0.023)
2010×Outlet entry before 2006 ($\theta^{outlet}_{1,2010}$)	0.007	-0.016	0.044**	0.046***	0.014	-0.024
	(0.016)	(0.024)	(0.018)	(0.014)	(0.021)	(0.022)
2015×Outlet entry before 2006 ($\theta^{outlet}_{1,2015}$)	0.001	-0.025	0.008	0.016	0.030	0.029
	(0.019)	(0.023)	(0.021)	(0.020)	(0.021)	(0.036)
2010×Outlet entry in 2006/10 ($\theta^{outlet}_{2,2010}$)	-0.017**	-0.034	-0.015	-0.005	-0.002	0.007
	(0.007)	(0.023)	(0.029)	(0.025)	(0.018)	(0.016)
2015×Outlet entry in 2006/10 ($\theta^{outlet}_{2,2015}$)	0.031***	-0.027	-0.001	0.028	0.012	0.009
	(0.005)	(0.020)	(0.024)	(0.020)	(0.018)	(0.015)
2015×Outlet entry in 2010/15 ($\theta^{outlet}_{3,2015}$)	0.003	-0.014	-0.018	-0.004	0.005	-0.007
	(0.053)	(0.033)	(0.024)	(0.019)	(0.022)	(0.022)
**Pre-treatment trends:**						
2010×Supermarket entry in 2010/15 ($\theta^{super}_{3,2010}$)	-0.022	-0.026	-0.027	-0.027	0.002	-0.038
	(0.022)	(0.026)	(0.023)	(0.019)	(0.029)	(0.033)
2010×Outlet entry in 2010/15 ($\theta^{outlet}_{3,2010}$)	-0.053***	-0.017	-0.003	-0.000	-0.000	-0.012
	(0.020)	(0.018)	(0.012)	(0.011)	(0.017)	(0.016)
Observations	3,709	3,624	3,309	3,160	2,925	2,497
R^2	0.910	0.911	0.912	0.913	0.911	0.914

NOTES: Estimates of θ based on the estimation of equation (1) using alternative measures of main competitors. Columns 1 to 4 take the main competitors of pharmacy i as its N nearest neighbors, with $N = 1, 2, 4, 5$, respectively. Columns 5 and 6 consider all retailers located within a radius of 600 and 800 meters, respectively, as main competitors. All models include year, drug, and pharmacy fixed-effects. Standard errors shown in parenthesis are clustered at the pharmacy level. $*p < 0.10, **p < 0.05, ***p < 0.01$.

Table S4.4: Results from estimating equation (1) among the most spatially isolated pharmacies in 2006

	Number of nearest neighbors				Radius	
	1	2	4	5	600m	800m
	(1)	(2)	(3)	(4)	(5)	(6)
**DID estimates:**						
2010×Supermarket entry before 2006 ($\theta^{super}_{1,2010}$)		-0.032***	-0.034***		-0.042***	
		(0.008)	(0.009)		(0.009)	
2015×Supermarket entry before 2006 ($\theta^{super}_{1,2015}$)		-0.025***	-0.020*		-0.027**	
		(0.008)	(0.010)		(0.011)	
2010×Supermarket entry in 2006/10 ($\theta^{super}_{2,2010}$)		-0.070***	-0.102***		-0.059**	-0.058**
		(0.009)	(0.020)		(0.027)	(0.027)
2015×Supermarket entry in 2006/10 ($\theta^{super}_{2,2015}$)	-0.058**	-0.116***	-0.077***	-0.55*	-0.049**	-0.043**
	(0.028)	(0.010)	(0.021)	(0.032)	(0.022)	(0.019)
2015×Supermarket entry in 2010/15 ($\theta^{super}_{3,2015}$)	-0.061**	-0.045***	-0.029*	-0.056**	-0.038	0.040*
	(0.029)	(0.016)	(0.016)	(0.025)	(0.034)	(0.023)
2010×Outlet entry before 2006 ($\theta^{outlet}_{1,2010}$)	-0.029	0.035***	0.028**	-0.055**	-0.006	0.030*
	(0.027)	(0.013)	(0.014)	(0.025)	(0.030)	(0.018)
2015×Outlet entry before 2006 ($\theta^{outlet}_{1,2015}$)	0.037**	0.019	-0.012	0.031**	-0.027**	0.096
	(0.016)	(0.031)	(0.016)	(0.013)	(0.013)	(0.062)
2010×Outlet entry in 2006/10 ($\theta^{outlet}_{2,2010}$)	0.032	-0.025	-0.023	-0.005	-0.050**	0.001
	(0.040)	(0.026)	(0.028)	(0.034)	(0.023)	(0.028)
2015×Outlet entry in 2006/10 ($\theta^{outlet}_{2,2015}$)	-0.012	-0.015	-0.006	0.023	-0.017	0.028
	(0.009)	(0.024)	(0.027)	(0.031)	(0.025)	(0.022)
2015×Outlet entry in 2010/15 ($\theta^{outlet}_{3,2015}$)	0.131***	0.059	-0.093***	-0.067*	0.020	-0.070
	(0.009)	(0.052)	(0.026)	(0.035)	(0.077)	(0.047)
**Pre-treatment trends:**						
2010×Supermarket entry in 2010/15 ($\theta^{super}_{3,2010}$)	-0.040***	-0.035	-0.040	-0.050	-0.050	-0.033
	(0.012)	(0.037)	(0.028)	(0.047)	(0.064)	(0.042)
2010×Outlet entry in 2010/15 ($\theta^{outlet}_{3,2010}$)	-0.091***	-0.014	-0.010	-0.032	-0.034	-0.035***
	(0.008)	(0.037)	(0.009)	(0.032)	(0.056)	(0.012)
Observations	1,288	1,292	1,137	903	933	733
R^2	0.916	0.919	0.921	0.921	0.914	0.922

NOTES: Estimates of θ based on the estimation of equation (1) among pharmacies located in areas where market structure is the most concentrated (ie. closest to a monopoly). Columns 1 to 4 take the main competitors of pharmacy i as its N nearest neighbors, with $N = 1, 2, 4, 5$, respectively. Columns 5 and 6 consider all retailers located within a radius of 600 and 800 meters, respectively, as main competitors. In columns 1 to 4 the samples were restricted to pharmacies whose walking time (in minutes) to their Nth nearest neighbor is above the sample mean in 2006. In columns 5 and 6 the samples were restricted to pharmacies whose number of competitors within the relevant radius in 2006 is below the sample median for the relevant radius distance. All models include year, drug, and pharmacy fixed-effects. Standard errors shown in parenthesis are clustered at the pharmacy level. $*p < 0.10, **p < 0.05, ***p < 0.01$.

Table S4.5: Results from estimating equation (1) among the least spatially isolated pharmacies in 2006

	Number of nearest neighbors				Radius	
	1	2	4	5	600m	800m
	(1)	(2)	(3)	(4)	(5)	(6)
**DID estimates:**						
2010×Supermarket entry before 2006 ($\theta^{super}_{1,2010}$)						
2015×Supermarket entry before 2006 ($\theta^{super}_{1,2015}$)						
2010×Supermarket entry in 2006/10 ($\theta^{super}_{2,2010}$)			-0.086**		0.040	0.008
			(0.034)		(0.036)	(0.074)
2015×Supermarket entry in 2006/10 ($\theta^{super}_{2,2015}$)			-0.028		-0.044	0.015
			(0.025)		(0.038)	(0.038)
2015×Supermarket entry in 2010/15 ($\theta^{super}_{3,2015}$)	-0.048***	-0.031	-0.032	-0.019	-0.025	-0.056
	(0.009)	(0.023)	(0.022)	(0.022)	(0.031)	(0.050)
2010×Outlet entry before 2006 ($\theta^{outlet}_{1,2010}$)	-0.036*	-0.041	0.047**	0.065***	0.025	-0.023
	(0.018)	(0.032)	(0.020)	(0.021)	(0.033)	(0.023)
2015×Outlet entry before 2006 ($\theta^{outlet}_{1,2015}$)	-0.004	-0.053**	0.013	0.050*	0.062***	0.019
	(0.009)	(0.021)	(0.023)	(0.026)	(0.019)	(0.038)
2010×Outlet entry in 2006/10 ($\theta^{outlet}_{2,2010}$)		-0.057	-0.011	0.018	0.042*	0.009
		(0.036)	(0.042)	(0.031)	(0.024)	(0.021)
2015×Outlet entry in 2006/10 ($\theta^{outlet}_{2,2015}$)		-0.078***	-0.018	0.043*	0.037	-0.014
		(0.019)	(0.035)	(0.024)	(0.026)	(0.022)
2015×Outlet entry in 2010/15 ($\theta^{outlet}_{3,2015}$)	-0.072***	-0.054	-0.023	0.001	0.001	0.008
	(0.009)	(0.034)	(0.024)	(0.023)	(0.024)	(0.024)
**Pre-treatment trends:**						
2010×Supermarket entry in 2010/15 ($\theta^{super}_{3,2010}$)	0.053***	0.026*	-0.021	0.018	0.003	-0.042
	(0.009)	(0.013)	(0.021)	(0.025)	(0.040)	(0.055)
2010×Outlet entry in 2010/15 ($\theta^{outlet}_{3,2010}$)	-0.013	-0.018	-0.005	0.006	0.008	0.012
	(0.009)	(0.020)	(0.012)	(0.015)	(0.020)	(0.020)
Observations	1,102	1,497	1,856	1,099	924	760
R^2	0.908	0.915	0.919	0.919	0.925	0.926

NOTES: Estimates of θ based on the estimation of equation (1) among pharmacies located in areas where market structure is the least concentrated (ie. furthest from a monopoly). Columns 1 to 4 take the main competitors of pharmacy i as its N nearest neighbors, with $N = 1, 2, 4, 5$, respectively. Columns 5 and 6 consider all retailers located within a radius of 600 and 800 meters, respectively, as main competitors. In columns 1 to 4 the samples were restricted to pharmacies whose walking time (in minutes) to their Nth nearest neighbor is below the sample mean in 2006. In columns 5 and 6 the samples were restricted to pharmacies whose number of competitors within the relevant radius in 2006 is above the sample median for the relevant radius distance. All models include year, drug, and pharmacy fixed-effects. Standard errors shown in parenthesis are clustered at the pharmacy level. $*p < 0.10, **p < 0.05, ***p < 0.01$.

Table S4.6: Results from estimating equation (1) among all retailer types

	Number of nearest neighbors				Radius	
	1	2	4	5	600m	800m
	(1)	(2)	(3)	(4)	(5)	(6)
**DID estimates:**						
2010×Supermarket entry before 2006 ($\theta^{super}_{1,2010}$)		-0.039***	-0.041***		-0.036***	
		(0.006)	(0.006)		(0.007)	
2015×Supermarket entry before 2006 ($\theta^{super}_{1,2015}$)		-0.020***	-0.022***		-0.020***	
		(0.006)	(0.006)		(0.008)	
2010×Supermarket entry in 2006/10 ($\theta^{super}_{2,2010}$)	-0.064**	-0.096***	-0.123***	-0.053*	-0.022	-0.037
	(0.027)	(0.016)	(0.037)	(0.031)	(0.026)	(0.032)
2015×Supermarket entry in 2006/10 ($\theta^{super}_{2,2015}$)	-0.058**	-0.092***	-0.107*	-0.042*	-0.038*	-0.030
	(0.028)	(0.017)	(0.056)	(0.023)	(0.020)	(0.023)
2015×Supermarket entry in 2010/15 ($\theta^{super}_{3,2015}$)	-0.032	-0.034***	-0.027	-0.026*	-0.010	-0.045*
	(0.021)	(0.012)	(0.018)	(0.015)	(0.021)	(0.023)
2010×Outlet entry before 2006 ($\theta^{outlet}_{1,2010}$)	0.006	-0.025	0.012	0.043***	0.014	-0.024
	(0.016)	(0.024)	(0.030)	(0.014)	(0.021)	(0.022)
2015×Outlet entry before 2006 ($\theta^{outlet}_{1,2015}$)	0.004	-0.018	-0.004	0.018	0.030	0.029
	(0.019)	(0.023)	(0.024)	(0.020)	(0.021)	(0.036)
2010×Outlet entry in 2006/10 ($\theta^{outlet}_{2,2010}$)	-0.003	-0.029	-0.019	-0.004	-0.002	0.007
	(0.014)	(0.021)	(0.029)	(0.024)	(0.018)	(0.016)
2015×Outlet entry in 2006/10 ($\theta^{outlet}_{2,2015}$)	0.041***	-0.020	0.001	0.032*	0.012	0.009
	(0.009)	(0.020)	(0.024)	(0.019)	(0.018)	(0.015)
2015×Outlet entry in 2010/15 ($\theta^{outlet}_{3,2015}$)	0.006	-0.010	-0.016	-0.002	0.005	-0.007
	(0.053)	(0.033)	(0.024)	(0.020)	(0.022)	(0.022)
**Pre-treatment trends:**						
2010×Supermarket entry in 2010/15 ($\theta^{super}_{3,2010}$)	-0.023	-0.027	-0.031	-0.030	-0.000	-0.038
	(0.022)	(0.026)	(0.023)	(0.019)	(0.017)	(0.033)
2010×Outlet entry in 2010/15 ($\theta^{outlet}_{3,2010}$)	-0.054***	-0.018	-0.006	-0.004	0.008	-0.012
	(0.020)	(0.018)	(0.012)	(0.012)	(0.020)	(0.016)
Observations	4,141	4,056	3,716	3,542	2,925	2,497
R^2	0.904	0.904	0.905	0.906	0.911	0.914

NOTES: Estimates of θ based on the estimation of equation (1) among all retailer types: traditional pharmacies, supermarkets and outlets. Columns 1 to 4 take the main competitors of pharmacy i as its N nearest neighbors, with $N = 1, 2, 4, 5$, respectively. Columns 5 and 6 consider all retailers located within a radius of 600 and 800 meters, respectively, as main competitors. All models include year, drug, and pharmacy fixed-effects. Standard errors shown in parenthesis are clustered at the retailer level. $*p < 0.10, **p < 0.05, ***p < 0.01$.

Table S4.7: Results from estimating equation (1) among pharmacies whose competitors are all in the control group

	Number of nearest neighbors				Radius	
	1	2	4	5	600m	800m
	(1)	(2)	(3)	(4)	(5)	(6)
**DID estimates:**						
2010×Supermarket entry before 2006 ($\theta^{super}_{1,2010}$)		-0.036***	-0.054***		-0.030***	
		(0.006)	(0.019)		(0.009)	
2015×Supermarket entry before 2006 ($\theta^{super}_{1,2015}$)		-0.022***	-0.033*		-0.022**	
		(0.006)	(0.018)		(0.010)	
2010×Supermarket entry in 2006/10 ($\theta^{super}_{2,2010}$)	-0.064**	-0.093***	-0.059***	-0.048	-0.017	-0.038
	(0.027)	(0.017)	(0.022)	(0.032)	(0.026)	(0.034)
2015×Supermarket entry in 2006/10 ($\theta^{super}_{2,2015}$)	-0.063**	-0.094***	-0.057**	-0.048**	-0.040*	-0.034
	(0.028)	(0.018)	(0.025)	(0.024)	(0.021)	(0.026)
2015×Supermarket entry in 2010/15 ($\theta^{super}_{3,2015}$)	-0.037*	-0.036***	-0.005	-0.031**	-0.012	-0.049*
	(0.021)	(0.013)	(0.020)	(0.016)	(0.022)	(0.026)
2010×Outlet entry before 2006 ($\theta^{outlet}_{1,2010}$)	0.006	-0.015	0.024	0.047***	0.020	-0.025
	(0.016)	(0.024)	(0.021)	(0.016)	(0.021)	(0.024)
2015×Outlet entry before 2006 ($\theta^{outlet}_{1,2015}$)	-0.001	-0.024	0.010	0.013	0.028	0.025
	(0.019)	(0.023)	(0.021)	(0.021)	(0.022)	(0.038)
2010×Outlet entry in 2006/10 ($\theta^{outlet}_{2,2010}$)	-0.018**	-0.033	-0.012	-0.004	0.004	0.006
	(0.007)	(0.023)	(0.030)	(0.025)	(0.019)	(0.020)
2015×Outlet entry in 2006/10 ($\theta^{outlet}_{2,2015}$)	0.029***	-0.025	0.003	0.024	0.010	0.005
	(0.006)	(0.021)	(0.024)	(0.025)	(0.018)	(0.020)
2015×Outlet entry in 2010/15 ($\theta^{outlet}_{3,2015}$)	0.001	-0.013	0.001	-0.008	0.003	-0.013
	(0.053)	(0.034)	(0.020)	(0.020)	(0.022)	(0.020)
**Pre-treatment trends:**						
2010×Supermarket entry in 2010/15 ($\theta^{super}_{3,2010}$)	-0.023	-0.025	-0.025	-0.025	0.008	-0.039
	(0.022)	(0.026)	(0.015)	(0.015)	(0.029)	(0.035)
2010×Outlet entry in 2010/15 ($\theta^{outlet}_{3,2010}$)	-0.053***	-0.015	0.001	0.001	0.006	0.005
	(0.020)	(0.018)	(0.013)	(0.013)	(0.018)	(0.020)
Observations	3,246	2,849	1,858	1,712	1,875	1,486
R^2	0.911	0.910	0.912	0.918	0.913	0.909

NOTES: Estimates of θ based on the estimation of equation (1) among pharmacies whose competitors are all in the control group. Columns 1 to 4 take the main competitors of pharmacy i as its N nearest neighbors, with $N = 1, 2, 4, 5$, respectively. Columns 5 and 6 consider all retailers located within a radius of 600 and 800 meters, respectively, as main competitors. All models include year, drug, and pharmacy fixed-effects. Standard errors shown in parenthesis are clustered at the pharmacy level. $*p < 0.10, **p < 0.05, ***p < 0.01$.

Table S4.8: Results from estimating equation (1) in a balanced panel of pharmacies

	Number of nearest neighbors				Radius	
	1	2	4	5	600m	800m
	(1)	(2)	(3)	(4)	(5)	(6)
**DID estimates:**						
2010×Supermarket entry before 2006 ($\theta^{super}_{1,2010}$)		-0.034***	-0.052***		-0.031***	
		(0.005)	(0.019)		(0.007)	
2015×Supermarket entry before 2006 ($\theta^{super}_{1,2015}$)		-0.023***	0.033*		-0.019**	
		(0.006)	(0.018)		(0.009)	
2010×Supermarket entry in 2006/10 ($\theta^{super}_{2,2010}$)	-0.060**	-0.090***	-0.057***	-0.041	-0.035	-0.033
	(0.027)	(0.016)	(0.021)	(0.031)	(0.022)	(0.029)
2015×Supermarket entry in 2006/10 ($\theta^{super}_{2,2015}$)	-0.060**	-0.095***	-0.057**	-0.041*	-0.045**	-0.029
	(0.028)	(0.018)	(0.024)	(0.024)	(0.018)	(0.021)
2015×Supermarket entry in 2010/15 ($\theta^{super}_{3,2015}$)	-0.034*	-0.034***	-0.004	-0.023	-0.017	-0.044*
	(0.021)	(0.012)	(0.019)	(0.015)	(0.021)	(0.023)
2010×Outlet entry before 2006 ($\theta^{outlet}_{1,2010}$)	0.010	-0.013	0.025	0.054***	0.032	-0.008
	(0.016)	(0.024)	(0.021)	(0.016)	(0.022)	(0.023)
2015×Outlet entry before 2006 ($\theta^{outlet}_{1,2015}$)	0.002	-0.024	0.008	0.019	0.037*	0.035
	(0.019)	(0.023)	(0.021)	(0.021)	(0.037)	
2010×Outlet entry in 2006/10 ($\theta^{outlet}_{2,2010}$)	-0.014*	-0.031	0.010	0.027	-0.012	-0.000
	(0.007)	(0.023)	(0.025)	(0.021)	(0.019)	(0.016)
2015×Outlet entry in 2006/10 ($\theta^{outlet}_{2,2015}$)	0.032***	-0.026	0.013	0.043**	0.006	0.003
	(0.006)	(0.021)	(0.023)	(0.019)	(0.018)	(0.015)
2015×Outlet entry in 2010/15 ($\theta^{outlet}_{3,2015}$)	0.005	-0.013	0.001	-0.001	0.006	-0.006
	(0.053)	(0.033)	(0.022)	(0.020)	(0.022)	(0.022)
**Pre-treatment trends:**						
2010×Supermarket entry in 2010/15 ($\theta^{super}_{3,2010}$)	-0.019	-0.018	-0.030	-0.017	0.000	-0.031
	(0.022)	(0.026)	(0.019)	(0.020)	(0.028)	(0.034)
2010×Outlet entry in 2010/15 ($\theta^{outlet}_{3,2010}$)	-0.049**	-0.013	0.009	0.009	0.005	-0.005
	(0.020)	(0.018)	(0.012)	(0.012)	(0.017)	(0.016)
Observations	2,460	2,385	2,235	1,923	1,698	1,314
R^2	0.912	0.920	0.921	0.924	0.922	0.928

NOTES: Estimates of θ based on the estimation of equation (1) among pharmacies who are observed at all time periods (2006, 2010, 2015). Columns 1 to 4 take the main competitors of pharmacy i as its N nearest neighbors, with $N = 1, 2, 4, 5$, respectively. Columns 5 and 6 consider all retailers located within a radius of 600 and 800 meters, respectively, as main competitors. All models include year, drug, and pharmacy fixed-effects. Standard errors shown in parenthesis are clustered at the pharmacy level. $*p < 0.10, **p < 0.05, ***p < 0.01$.

Table S4.9: Results from estimating equation (1) among pharmacies whose main competitors did not exit

	Number of nearest neighbors				Radius	
	1	2	4	5	600m	800m
	(1)	(2)	(3)	(4)	(5)	(6)
**DID estimates:**						
2010×Supermarket entry before 2006 ($\theta^{super}_{1,2010}$)		-0.035***	-0.032***			
		(0.006)	(0.008)			
2015×Supermarket entry before 2006 ($\theta^{super}_{1,2015}$)		-0.024***	-0.024***			
		(0.006)	(0.007)			
2010×Supermarket entry in 2006/10 ($\theta^{super}_{2,2010}$)	-0.060*	-0.103***	-0.126***	-0.040	-0.023	-0.039
	(0.036)	(0.019)	(0.008)	(0.038)	(0.026)	(0.032)
2015×Supermarket entry in 2006/10 ($\theta^{super}_{2,2015}$)	-0.041	-0.082***	-0.057***	-0.027	-0.039*	-0.031
	(0.030)	(0.019)	(0.007)	(0.024)	(0.020)	(0.023)
2015×Supermarket entry in 2010/15 ($\theta^{super}_{3,2015}$)	-0.034*	-0.040***	-0.050*	-0.049*	-0.017	-0.060**
	(0.021)	(0.014)	(0.027)	(0.026)	(0.022)	(0.023)
2010×Outlet entry before 2006 ($\theta^{outlet}_{1,2010}$)	0.011	0.046***			0.027	-0.025
	(0.031)	(0.014)			(0.021)	(0.022)
2015×Outlet entry before 2006 ($\theta^{outlet}_{1,2015}$)	-0.028	-0.045**			0.043**	0.028
	(0.017)	(0.023)			(0.020)	(0.036)
2010×Outlet entry in 2006/10 ($\theta^{outlet}_{2,2010}$)	-0.016**	-0.055*	-0.022	0.028	-0.003	0.005
	(0.007)	(0.031)	(0.036)	(0.030)	(0.018)	(0.016)
2015×Outlet entry in 2006/10 ($\theta^{outlet}_{2,2015}$)	0.032***	-0.010	0.012	0.055***	0.010	0.008
	(0.006)	(0.029)	(0.029)	(0.021)	(0.018)	(0.015)
2015×Outlet entry in 2010/15 ($\theta^{outlet}_{3,2015}$)	0.004	-0.022	-0.005	0.017	-0.008	-0.022
	(0.053)	(0.047)	(0.036)	(0.031)	(0.021)	(0.019)
**Pre-treatment trends:**						
2010×Treated with supermarket in 201015	-0.021	-0.042	-0.070**	-0.081***	-0.006	-0.053
	(0.022)	(0.046)	(0.029)	(0.029)	(0.030)	(0.033)
2010×Outlet entry in 2010/15 ($\theta^{outlet}_{3,2010}$)	-0.052***	-0.025	-0.010	0.001	-0.005	-0.018
	(0.020)	(0.020)	(0.013)	(0.016)	(0.018)	(0.017)
Observations	3,099	2,448	1,532	1,242	2,675	2,412
R^2	0.910	0.911	0.917	0.918	0.910	0.915

NOTES: Estimates of θ based on the estimation of equation (1) among pharmacies whose main competitors do not exit the market during the time horizon under analysis. Columns 1 to 4 take the main competitors of pharmacy i as its N nearest neighbors, with $N = 1, 2, 4, 5$, respectively. Columns 5 and 6 consider all retailers located within a radius of 600 and 800 meters, respectively, as main competitors. All models include year, drug, and pharmacy fixed-effects. Standard errors shown in parenthesis are clustered at the pharmacy level. $*p < 0.10, **p < 0.05, ***p < 0.01$.

Table S4.10: Results from estimating equation (1) with non-mutually exclusive treatments

	Number of nearest neighbors				Radius	
	1	2	4	5	600m	800m
	(1)	(2)	(3)	(4)	(5)	(6)
**DID estimates:**						
2010×Supermarket entry before 2006 ($\theta^{super}_{1,2010}$)		-0.038***	-0.036***	-0.032	-0.037***	-0.052***
		(0.005)	(0.006)	(0.024)	(0.007)	(0.014)
2015×Supermarket entry before 2006 ($\theta^{super}_{1,2015}$)		-0.023***	-0.021***	-0.049***	-0.021***	-0.038***
		(0.005)	(0.006)	(0.019)	(0.008)	(0.013)
2010×Supermarket entry in 2006/10 ($\theta^{super}_{2,2010}$)	-0.063**	-0.094***	-0.081***	-0.048	-0.023	-0.037
	(0.027)	(0.016)	(0.023)	(0.031)	(0.026)	(0.032)
2015×Supermarket entry in 2006/10 ($\theta^{super}_{2,2015}$)	-0.062**	-0.095***	-0.048*	-0.041*	-0.038*	-0.029
	(0.028)	(0.017)	(0.028)	(0.023)	(0.020)	(0.023)
2015×Supermarket entry in 2010/15 ($\theta^{super}_{3,2015}$)	-0.035*	-0.037***	-0.008	0.010	-0.013	-0.040*
	(0.020)	(0.012)	(0.020)	(0.015)	(0.018)	(0.022)
2010×Outlet entry before 2006 ($\theta^{outlet}_{1,2010}$)	0.007	-0.016	0.044**	0.048***	0.013	-0.023
	(0.016)	(0.024)	(0.018)	(0.014)	(0.021)	(0.022)
2015×Outlet entry before 2006 ($\theta^{outlet}_{1,2015}$)	0.001	-0.025	0.010	0.019	0.029	0.030
	(0.019)	(0.023)	(0.021)	(0.020)	(0.021)	(0.036)
2010×Outlet entry in 2006/10 ($\theta^{outlet}_{2,2010}$)	-0.017**	-0.034	-0.015	-0.004	-0.003	0.007
	(0.007)	(0.023)	(0.029)	(0.025)	(0.018)	(0.016)
2015×Outlet entry in 2006/10 ($\theta^{outlet}_{2,2015}$)	0.031***	-0.027	0.001	0.031	0.012	0.010
	(0.005)	(0.020)	(0.024)	(0.020)	(0.017)	(0.015)
2015×Outlet entry in 2010/15 ($\theta^{outlet}_{3,2015}$)	0.003	-0.014	0.001	0.014	0.002	-0.003
	(0.053)	(0.033)	(0.023)	(0.018)	(0.019)	(0.020)
**Pre-treatment trends:**						
2010×Supermarket entry in 2010/15 ($\theta^{super}_{3,2010}$)	-0.022	-0.026	-0.027	-0.022	-0.007	-0.034
	(0.022)	(0.026)	(0.020)	(0.017)	(0.021)	(0.023)
2010×Outlet entry in 2010/15 ($\theta^{outlet}_{3,2010}$)	-0.053***	-0.014	-0.003	0.004	-0.009	-0.008
	(0.020)	(0.033)	(0.012)	(0.012)	(0.016)	(0.017)
Observations	3,769	3,769	3,769	3,769	3,769	3,769
R^2	0.908	0.909	0.909	0.909	0.909	0.909

NOTES: Estimates of θ based on the estimation of equation (1) among pharmacies, without imposing mutually exclusivity of treatment groups. Columns 1 to 4 take the main competitors of pharmacy i as its N nearest neighbors, with $N = 1, 2, 4, 5$, respectively. Columns 5 and 6 consider all retailers located within a radius of 600 and 800 meters, respectively, as main competitors. All models include year, drug, and pharmacy fixed-effects as well as interactions between different treatment groups. Standard errors shown in parenthesis are clustered at the pharmacy level. $*p < 0.10, **p < 0.05, ***p < 0.01$.

Table S4.11: Results from estimating equation (1) with 2-way clustering of standard errors by drug and pharmacy

	Number of nearest neighbors (N)				Radius	
	1	2	4	5	600m	800m
**DiD estimates:**						
2010×Supermarket entry before 2006 ($\theta^{super}_{1,2010}$)		-0.038**	-0.037**		-0.036**	
		(0.010)	(0.011)		(0.010)	
2015×Supermarket entry before 2006 ($\theta^{super}_{1,2015}$)		-0.023	-0.023		-0.020	
		(0.027)	(0.028)		(0.030)	
2010×Supermarket entry in 2006/10 ($\theta^{super}_{2,2010}$)	-0.063**	-0.094***	-0.082***	-0.049	-0.022	-0.037
	(0.017)	(0.015)	(0.018)	(0.027)	(0.026)	(0.029)
2015×Supermarket entry in 2006/10 ($\theta^{super}_{2,2015}$)	-0.062**	-0.095***	-0.050	-0.044**	-0.038**	-0.030*
	(0.016)	(0.007)	(0.024)	(0.011)	(0.009)	(0.013)
2015×Supermarket entry in 2010/15 ($\theta^{super}_{3,2015}$)	-0.035	-0.037***	-0.029**	-0.028*	-0.010	-0.045**
	(0.040)	(0.008)	(0.009)	(0.010)	(0.016)	(0.016)
2010×Outlet entry before 2006 ($\theta^{outlet}_{1,2010}$)	0.007	-0.016	0.044**	0.046**	0.014	-0.024
	(0.012)	(0.021)	(0.011)	(0.016)	(0.021)	(0.016)
2015×Outlet entry before 2006 ($\theta^{outlet}_{1,2015}$)	0.001	-0.025	0.008	0.016	0.030	0.029
	(0.011)	(0.018)	(0.012)	(0.015)	(0.022)	(0.033)
2010×Outlet entry in 2006/10 ($\theta^{outlet}_{2,2010}$)	-0.017**	-0.034	-0.015	-0.005	-0.002	0.007
	(0.009)	(0.022)	(0.029)	(0.028)	(0.014)	(0.018)
2015×Outlet entry in 2006/10 ($\theta^{outlet}_{2,2015}$)	0.031*	-0.027**	-0.001	0.028	0.012	0.009
	(0.014)	(0.009)	(0.020)	(0.017)	(0.012)	(0.008)
2015×Outlet entry in 2010/15 ($\theta^{outlet}_{3,2015}$)	0.003	-0.014	-0.018	-0.004	0.005	-0.007
	(0.054)	(0.031)	(0.022)	(0.017)	(0.017)	(0.021)
**Pre-treatment trends:**						
2010×Supermarket entry in 2010/15 ($\theta^{super}_{3,2010}$)	-0.022	-0.026	-0.027	-0.027	0.002	-0.038
	(0.020)	(0.022)	(0.020)	(0.017)	(0.026)	(0.030)
2010×Outlet entry in 2010/15 ($\theta^{outlet}_{3,2010}$)	-0.053*	-0.017	-0.003	-0.000	-0.000	-0.012
	(0.024)	(0.021)	(0.009)	(0.008)	(0.014)	(0.006)
Observations	3,709	3,624	3,309	3,160	2,925	2,497
R^2	0.910	0.911	0.912	0.913	0.918	0.921

NOTES: Estimates of θ based on the estimation of equation (1) among traditional pharmacies. Columns 1 to 4 take the main competitors of pharmacy i are its N nearest neighbors, with $N = 1, 2, 4, 5$, respectively. Columns 5 and 6 consider all retailers located within a radius of 600 and 800 meters, respectively, as main competitors. All specifications include year, drug, and pharmacy fixed-effects. Standard errors are shown in parenthesis are clustered at the pharmacy and drug level. * $p < 0.1$, ** $p < 0.05$, *** $p < 0.01$

Table S4.12: Results from estimating equation (1) in PS-matched samples of pharmacies using single neighbor matching

	Number of nearest neighbors (N)				Radius (R)	
	1	2	4	5	600m	800m
	(1)	(2)	(3)	(4)	(5)	(6)
**DID estimates:**						
2010×Supermarket entry in 2006/10 ($\theta^{super}_{2,2010}$)	-0.060***	-0.079***	-0.037*	-0.048	-0.007	-0.045
	(0.022)	(0.025)	(0.020)	(0.041)	(0.029)	(0.030)
2015×Supermarket entry in 2006/10 ($\theta^{super}_{2,2015}$)	-0.055***	-0.094***	-0.038	-0.048***	-0.027	-0.036
	(0.020)	(0.027)	(0.025)	(0.018)	(0.024)	(0.024)
2015×Supermarket entry in 2010/15 ($\theta^{super}_{3,2015}$)	-0.029*	-0.036***	-0.017	-0.032	-0.001	-0.039
	(0.015)	(0.008)	(0.015)	(0.020)	(0.029)	(0.028)
2010×Outlet entry in 2006/10 ($\theta^{outlet}_{2,2010}$)	-0.015**	-0.020	0.029	-0.004	0.017	-0.003
	(0.007)	(0.021)	(0.021)	(0.027)	(0.023)	(0.022)
2015×Outlet entry in 2006/10 ($\theta^{outlet}_{2,2015}$)	0.037**	-0.025	0.011	0.025	0.025	0.016
	(0.018)	(0.020)	(0.020)	(0.022)	(0.022)	(0.024)
2015×Outlet entry in 2010/15 ($\theta^{outlet}_{3,2015}$)	0.009	-0.012	-0.012	-0.005	0.004	-0.015
	(0.062)	(0.025)	(0.022)	(0.021)	(0.025)	(0.025)
**Pre-treatment trends:**						
2010×Supermarket entry in 2010/15 ($\theta^{super}_{3,2010}$)	-0.020	-0.011	0.017	-0.025	0.013	-0.051*
	(0.018)	(0.025)	(0.020)	(0.023)	(0.030)	(0.028)
2010×Outlet entry in 2010/15 ($\theta^{outlet}_{3,2010}$)	-0.050**	-0.002	0.039***	0.001	0.012	-0.029
	(0.023)	(0.014)	(0.007)	(0.021)	(0.027)	(0.021)
Observations	390	830	1,090	1,600	1,400	1,310
R^2	0.916	0.905	0.903	0.913	0.905	0.933

NOTES: Estimates of θ based on the estimation of equation (1) on a matched sample of pharmacies, with matching done on propensity scores using single nearest-neighbor matching. Columns 1 to 4 take the main competitors of pharmacy i as its N nearest neighbors, with $N = 1, 2, 4, 5$, respectively. Columns 5 and 6 consider all retailers located within a radius of 600 and 800 meters, respectively, as main competitors. All models include year, drug, and pharmacy fixed-effects. Standard errors shown in parenthesis are bootstrapped using 30 repetitions, drawn cross-sectionally at the pharmacy level in the original sample. $*p < 0.10, **p < 0.05, ***p < 0.01$.

Table S4.13: Results from estimating equation (1) in PS-matched samples of pharmacies using local linear regression

	Number of nearest neighbors (N)				Radius (R)	
	1	2	4	5	600m	800m
	(1)	(2)	(3)	(4)	(5)	(6)
**DID estimates:**						
2010×Supermarket entry in 2006/10 ($\theta^{super}_{2,2010}$) -0.060	-0.074**	-0.039	-0.034	-0.014	-0.047	
	(0.056)	(0.037)	(0.034)	(0.041)	(0.030)	(0.031)
2015×Supermarket entry in 2006/10 ($\theta^{super}_{2,2015}$)	-0.055	-0.092	-0.032	-0.040**	-0.027	-0.044*
	(0.067)	(0.057)	(0.037)	(0.019)	(0.025)	(0.026)
2015×Supermarket entry in 2010/15 ($\theta^{super}_{3,2015}$)	-0.029	-0.034	0.004	-0.024	-0.001	-0.047*
	(0.065)	(0.043)	(0.033)	(0.018)	(0.026)	(0.028)
2010×Outlet entry in 2006/10 ($\theta^{outlet}_{2,2010}$)	-0.015	-0.014	0.015	0.003	0.026	-0.006
	(0.050)	(0.053)	(0.058)	(0.026)	(0.024)	(0.023)
2015×Outlet entry in 2006/10 ($\theta^{outlet}_{2,2015}$)	0.037	-0.024	0.012	0.034	0.030	0.009
	(0.051)	(0.041)	(0.039)	(0.021)	(0.022)	(0.024)
2015×Outlet entry in 2010/15 ($\theta^{outlet}_{3,2015}$)	0.009	-0.010	-0.017	0.002	0.004	-0.022
	(0.062)	(0.051)	(0.027)	(0.021)	(0.025)	(0.025)
**Pre-treatment trends:**						
2010×Supermarket entry in 2010/15 ($\theta^{super}_{3,2010}$)	-0.020	-0.006	0.026	-0.011	0.013	-0.054*
	(0.052)	(0.046)	(0.037)	(0.024)	(0.030)	(0.028)
2010×Outlet entry in 2010/15 ($\theta^{outlet}_{3,2010}$)	-0.050	0.003	0.033	0.015	-0.012	-0.032
	(0.052)	(0.044)	(0.029)	(0.021)	(0.028)	(0.022)
Observations	390	830	1,030	1,560	1,390	1,580
R^2	0.916	0.905	0.904	0.915	0.905	0.930

NOTES: Estimates of θ based on the estimation of equation (1) on a matched sample of pharmacies, with matching done on propensity scores using local linear regression. Columns 1 to 4 take the main competitors of pharmacy i as its N nearest neighbors, with $N = 1, 2, 4, 5$, respectively. Columns 5 and 5 consider all retailers located within a radius of 600 and 800 meters, respectively, as main competitors. All models include year, drug, and pharmacy fixed-effects. Standard errors shown in parenthesis are bootstrapped using 30 repetitions, drawn cross-sectionally at the pharmacy level in the original sample. $*p < 0.10, **p < 0.05, ***p < 0.01$.

Table S4.14: Results from the estimation of the reduced-form entry model

Main Competitors	$\zeta(P_{t-1})$ specification	Supermarket	Outlet
Nearest neighbor	P_{it-1}	-0.716	-1.937*
		(1.647)	(1.033)
	P_{it-1} relatively to average market price	-0.744	-2.075**
		(1.525)	(1.042)
2 Nearest neighbors	P_{it-1}	-0.885	-0.154
		(5.882)	(0.649)
	P_{it-1} relatively to average market price	-0.802	-0.307
		(3.204)	(0.720)
4 Nearest neighbors	P_{it-1}	-0.912**	-0.593
		(0.448)	(0.917)
	P_{it-1} relatively to average market price	-1.200***	-0.793
		(0.421)	(1.537)
5 Nearest neighbors	P_{it-1}	-0.724	-0.082
		(0.615)	(0.681)
	P_{it-1} relatively to average market price	-0.903	-0.194
		(0.579)	(0.686)
600m radius	P_{it-1}	0.027	0.214
		(0.912)	(0.749)
	P_{it-1} relatively to average market price	-0.511	0.140
		(0.803)	(0.736)
800m radius	P_{it-1}	-0.490	-1.167
		(1.178)	(1.057)
	P_{it-1} relatively to average market price	-0.626	-1.242
		(1.068)	(1.034)

NOTES: Marginal effects of β_1 from RE logit estimation of equation (2), with dependent variable being an indicator for facing the entry of a supermarket (column 1) and an outlet (column 2). There are six panels, each corresponding to an alternative definition of main competitors of pharmacy i. In the top four panels, the main competitors of a pharmacy are its N nearest neighbors, with N=1,2,4,5, respectively. In the two bottom panels, the main competitors of a pharmacy are the retailers located within a radius of 600 and 800 meters, respectively. In each of the panels, the first row tests whether pharmacy i facing the entry of a supermarket/outlet among its main competitors depends on the prices it charged in the previous period, $\zeta(P_{t-1}) = P_{it-1}$. The corresponding figures can be interpreted as the percentage-point change in the probability of facing entry associated with a 1% higher OTC bundle price in the previous period. The second row tests whether it depends on the lagged prices of pharmacy i relatively to the average bundle price in the city of Lisbon. The corresponding figures can be interpreted as the percentage-point change associated with a 1-unit increase in the independent variable. Recall that our estimation sample differs according to how we define the set of main competitors of pharmacy i, so that a different number of observations is used to obtain each marginal effect shown on the table. Standard errors shown in parenthesis are clustered at the pharmacy level. $*p < 0.10, **p < 0.05, ***p < 0.01$.

S5 Additional Plots

Remark: In general, the plots do not suggest that supermarket or outlet entry systematically took place near pharmacies in the highest price deciles or the highest population deciles.

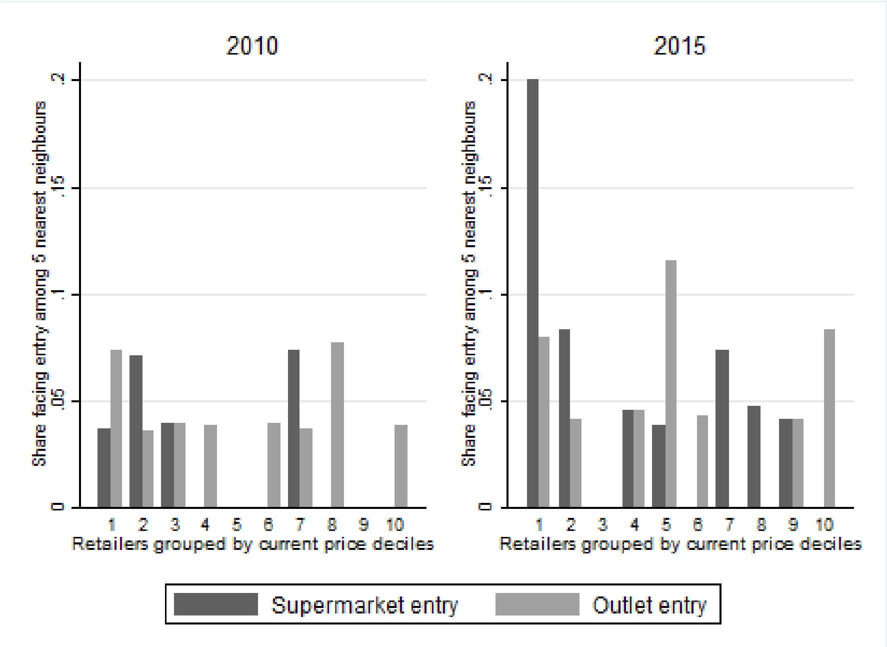

(a) By current price deciles

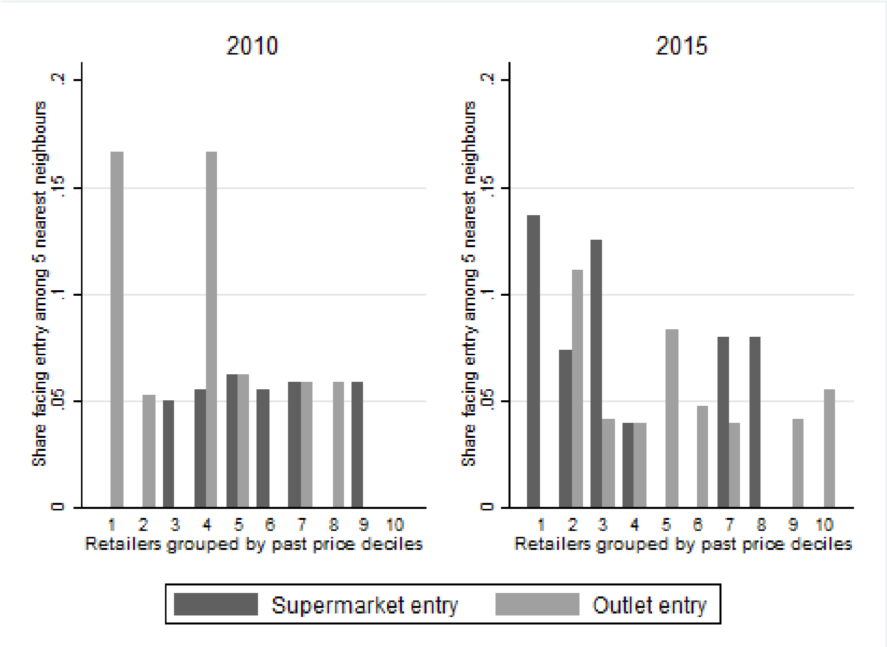

(b) By past price deciles

Figure S5.1: Share of pharmacies facing non-pharmacy entry among their 3 nearest neighbors, by price deciles

NOTES: In the top panel, pharmacies are grouped into deciles of their current price for the bundle of five OTC drugs considered in our analysis. In the bottom panel, pharmacies are grouped into deciles of their past price for the bundle of five OTC drugs considered in our analysis. In all the four plots the vertical axis indicates the share of pharmacies in each decile who faced the entry of a supermarket or outlet among their three nearest neighbors. We see that entry of supermarkets and outlets took place along all current and past price deciles in both 2010 and 2015, with no clear pattern.

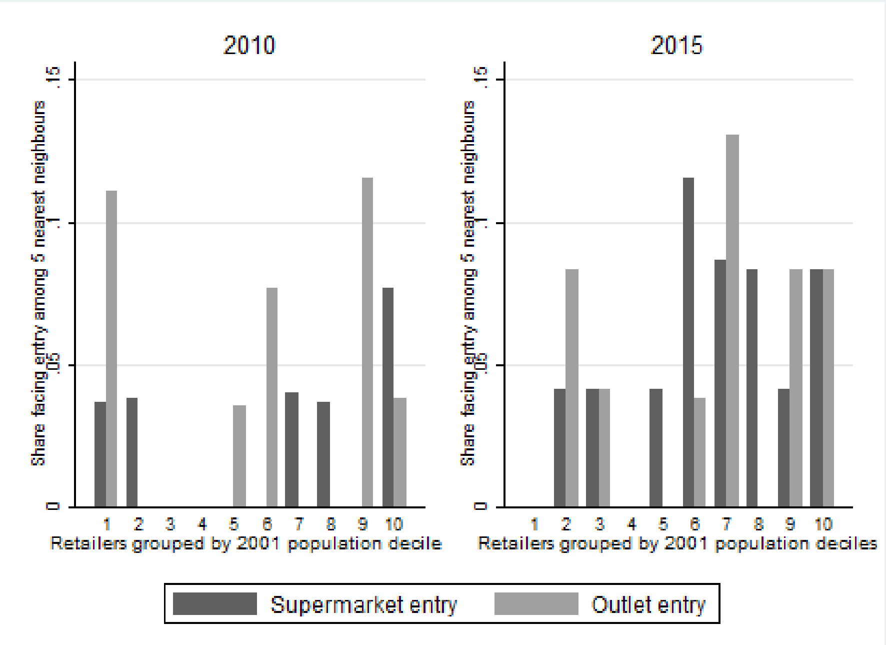

Figure S5.2: Share of pharmacies facing entry of non-pharmacies among the 3 nearest neighbors, by population deciles

NOTES: In order to create this figure, pharmacies are grouped into deciles of their 2001 level of demand, as measured by the resident population in the Census tract where they are located. In all the four plots the vertical axis indicates the share of pharmacies in each decile who faced the entry of a supermarket or outlet among their three nearest neighbors. We again see that entry of supermarkets and outlets took place along all population deciles in both 2010 and 2015, with no clear pattern.

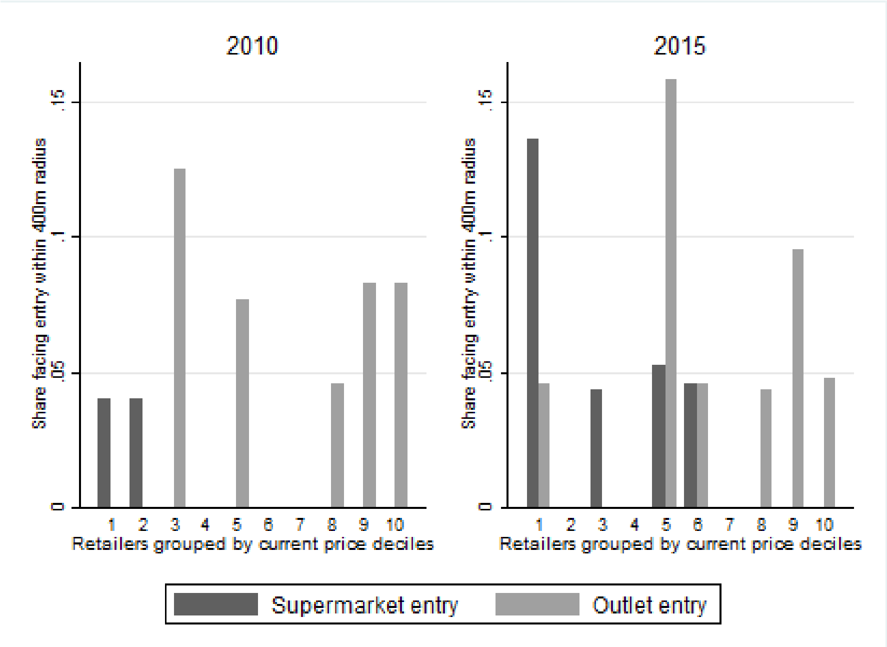

(a) By current price deciles

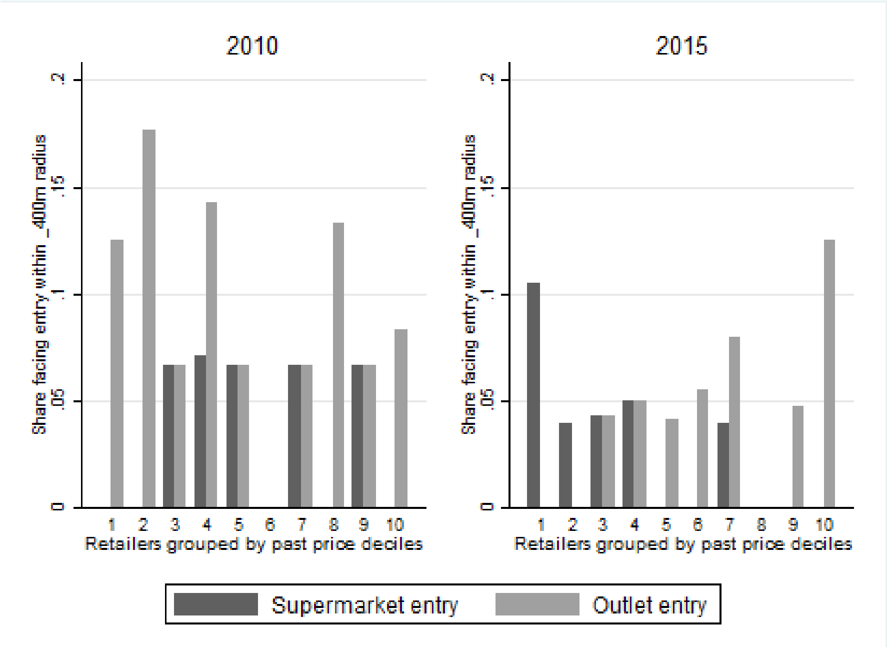

(b) By past price deciles

Figure S5.3: Share of pharmacies facing non-pharmacy entry within a 400-meter radius, by price deciles

NOTES: In the top panel, pharmacies are grouped into deciles of their current price for the bundle of five OTC drugs considered in our analysis. In the bottom panel, pharmacies are grouped into deciles of their past price for the bundle of five OTC drugs considered in our analysis. In all the four plots the vertical axis indicates the share of pharmacies in each decile who faced the entry of a supermarket or outlet within a 400-meter radius. We see that entry of supermarkets and outlets took place along all current and past price deciles in both 2010 and 2015, with no clear pattern.

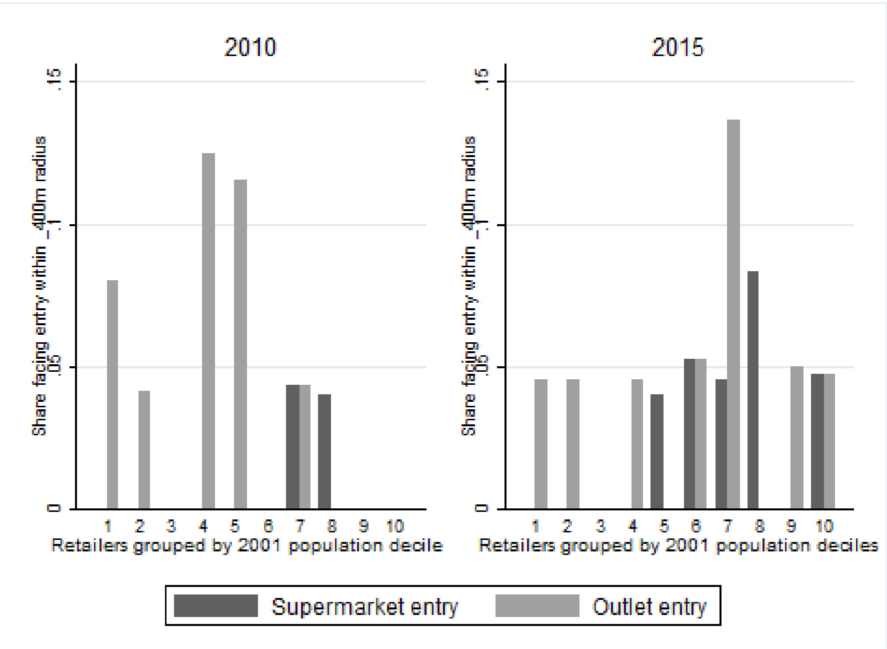

Figure S5.4: Share of pharmacies facing entry of non-pharmacies within a 400-meter radius, by population deciles

NOTES: In order to create this figure, pharmacies are grouped into deciles of their 2001 level of demand, as measured by the resident population in the Census tract where they are located. In all the four plots the vertical axis indicates the share of pharmacies in each decile who faced the entry of a supermarket or outlet within a 400-meter radius. We again see that entry of supermarkets and outlets took place along all population deciles in both 2010 and 2015, with no clear pattern.

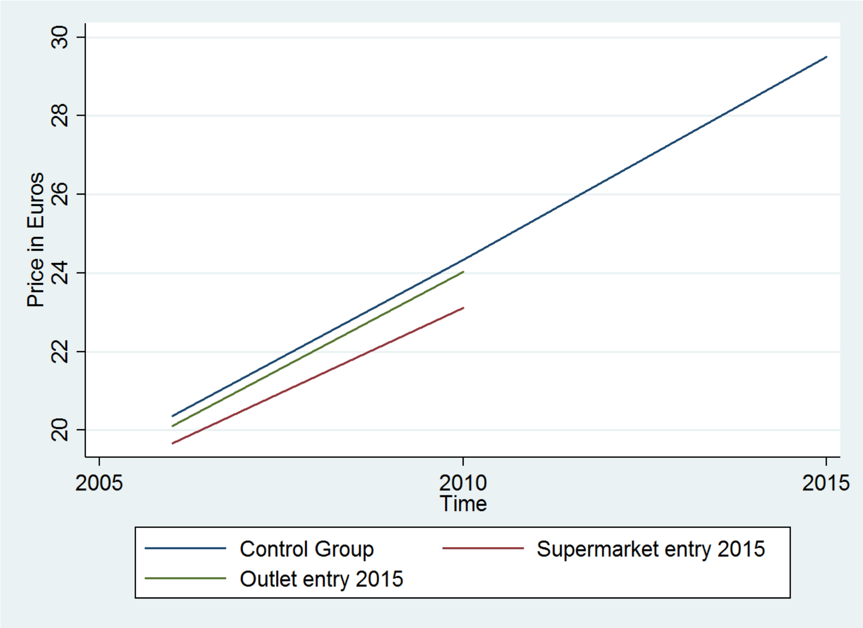

(a) $N = 3$

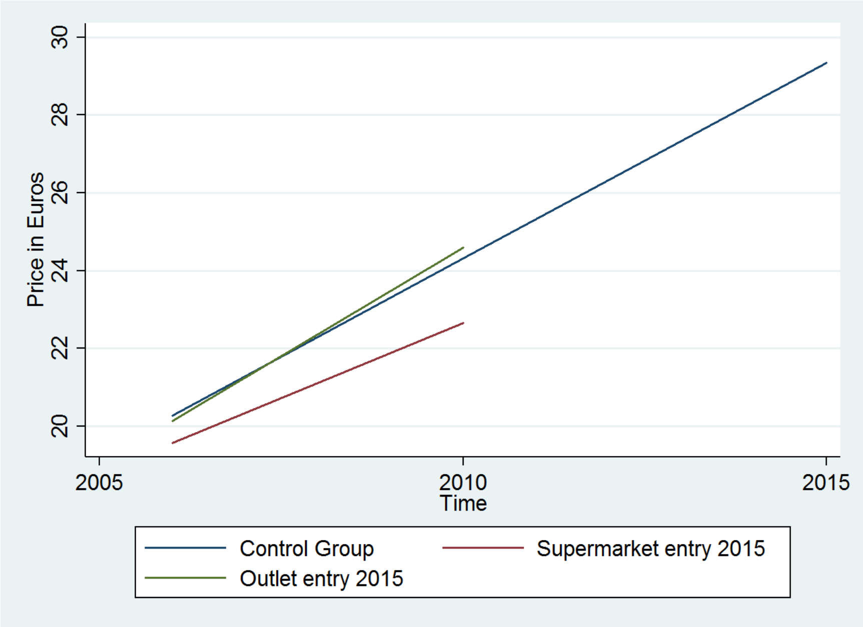

(b) 400-meter radius

Figure S5.5: Plot assessing the plausibility of the common trend assumption

NOTES: The figures compare the evolution of the price of the bundle of 5 drugs in our analysis for three distinct groups: the control group and the two groups of pharmacies who experienced entry of a supermarket or outlet among their main competitors after 2010. In panel (a) main competitors are the 3 nearest neighbors and in panel (b) they are all retailers located within a 400-meter radius. For groups treated after 2010 we observe prices for two pre-treatment periods and can compare their evolution with the control group. Overall, the plots do not suggest distinct price trends across the three groups between 2006 and 2010.

S6 Details on the PSM-DID procedure

We use a propensity score matching difference-in-differences approach to address the possible endogeneity of market structure (Heckman et al., 1997; Smith and Todd, 2005). The underlying intuition for this approach is that by matching treated and untreated pharmacies on their propensity score, that is, their probability of being treated, we make the groups more similar in terms of the observables used in the estimation of the propensity score. Thus, treatment should be random, conditional on the observables used to estimate the propensity score. The crucial assumption we are making with the use of PSM-DID is that, by achieving balancing on observables between the treated and control groups in the matched sample, it makes it more likely that such balancing also extends to unobservables, particularly time-variant unobservables (as time-invariant ones are in any case differenced out by the DID).

Below, we detail the more technical aspects regarding our implementation of PSM-DID.

Just like simple DID, PSM-DID yields estimates of the average treatment effect on the treated. PSM is, however, a data-demanding method. Typical applications of PSM control for a large set of observables in the estimation of the propensity score. While Heckman et al. (1997) shows that models using a richer set of covariates to estimate the propensity scores tend to be less biased, including more covariates also makes is more difficult to define the region of common support (Gibson-Davis and Foster, 2006). There is little guidance on how to balance this trade-off. As noted by Lechner (2010), one should include neither pre-treatment values of the outcome variable nor post-treatment values of independent variables in the estimation of the propensity score. With this in mind, and given that we do not have many variables available to estimate propensity scores, we opted for matching on few variables.

Specifically, we match pharmacies on two measures. These measures are the level of competitive pressure and the level of demand faced prior to experiencing non-pharmacy entry. Pre-entry levels of competitive pressure are measured as of 2006, our first data period. In the specifications using the N nearest neighbors as main competitors, pre-entry levels of competitive pressure are captured by the average walking time (in minutes) to the N nearest retailers in 2006. In the specifications using a radius distance to define the set of main competitors of a pharmacy, the pre-entry level of competitive pressure is given by the number of retailers within that radius in 2006. As for information of pre-entry levels of demand faced by each pharmacy, we complement our dataset with information from Statistics Portugal on the resident population in the Census tract where each pharmacy is located. This information was collected in the 2001 Census of the population.

We categorize the two variables used to estimate the propensity score into quintiles and we used the categorized variables for the matching. Given our unusual setting, featuring

multiple time periods and multiple treatments, we proceed as follows. Using a logit model, we estimate the propensity scores separately for each of the four treatment groups and for each year of our data. Therefore, for each model specification, a total of 12(=4 treatments $\times 3$ time periods) PSM procedures were carried out in order to obtain the matched sample of pharmacies. Given the estimated propensity scores, we match each treated pharmacy to its closest untreated PSM-neighbor at each time period (thus allowing us to easily accommodate some exit that we see in the data). We use two alternative methods for matching treated and untreated pharmacies. The first method is single nearest-neighbor within caliper matching with replacement, setting the caliper at 0.02. The second method consists of non-parametric local linear matching, with a bandwidth of 0.8.[4] Finally, we run our model specifications in this matched sample.

While asymptotically the estimates obtained should be independent of the matching method, this is not the case in small samples. In particular, nearest neighbor estimates may be the least biased, but are also less precise. Non-parametric methods, such as local linear regression, in turn, may be more biased, but have higher precision (Gibson-Davis and Foster, 2006). Therefore, if these two matched samples lead to similar price effects following the entry of supermarkets and outlets in the OTC market, then we have more confidence that these effects do not depend on the matching estimators used.

The standard errors of the estimates need to account for the propensity score estimation, the imputation of the common support, the fact that we are matching with replacement, and possibly also the order in which treated pharmacies are matched. A popular approach in this setting is to use bootstrapping methods. We bootstrap the entire procedure, meaning that we bootstrap pharmacies in the original sample, then carry out the estimation of the propensity scores and the matching procedure for each treatment and for each year, and finally estimate equation (1) in the matched sample for each of our bootstrapped samples.

We check covariate balancing between treatment and control groups in the original and matched samples. For the sake of brevity, and since 12 PSM procedures are carried out for each of the models we estimate, we do not show the results of covariate balancing tests or graphs of the common support condition. These are available upon request from the authors. In many, but nor all, of our PSM estimations we are able to achieve a decently balanced sample in terms of the covariates, and we thus assume that balance was achieved also in terms of unobservables.

Overall, the results of the PSM-DID are in line with those from the simple DID, though statistical significance is often lost. This may be a result of the smaller estimation samples used, as for each treated pharmacy we select only one matched untreated pharmacy.

[4]Different choices of caliper, number of neighbors matched, and bandwidth did not change our results.

S7 Overview of pharmacy regulations during 2005-2015

Table S7.1: Overview of pharmacy regulations during the period 2005-2015

Month & year	Legislation	Measures
August 2005	Decree-Law 134/2005	OTC drugs become available outside pharmacies.
February 2007	Ordinance 30B/2007	6% administrative price reduction for Government reimbursed drugs; Reduces margins for wholesalers and pharmacies.
March 2007	Decree-Law 60/2007	Introduces new rules for international price referencing to focus on low-price countries; Regulated drug prices become maximum prices and not fixed prices; Allows discounts at points of the value chain of pharmaceuticals in the ambulatory market (wholesale and retail), and sets a margin at each point.
June 2007	Decree-Law 238/2007	Enlarges the set of OTC drugs available outside pharmacies to include those subject to Government reimbursement; Reimbursement conditional on buying the drugs at a pharmacy.
August 2007	Decree-Law 307/2007	Liberalization of pharmacy ownership rules, with restrictions on the maximum number of pharmacies that can be owned by a single entity and on the professional categories that can own a pharmacy (ie. doctors, pharmaceutical companies, among others, cannot); Introduces exit restrictions (pharmacy opening restrictions were already in place).
November 2007	Ordinance 1430/2007	Changes the geographic criteria for the opening of new pharmacies by lowering number of inhabitants per pharmacy and the minimum distance between pharmacies.
October 2010	Ordinance 104-A/2010	6% administrative price reduction for Government reimbursed drugs.
January 2012	Decree-Law 112/2011	Introduces a new margin scheme for prescription drugs: there were changes in levels as well as the structure of the margins, with the introduction of a regressive margin. Also sets the price cap for the first generic entering the market to 50% of the price of the original drug.
May 2012	Ordinance 137-A/2012	Patients can substitute branded drugs for generics at the pharmacy; Pharmacies which must carry the 5 products with the lowest price in each reference group.
January 2013	Ordinance n 14/2013	Introduces some flexibility in terms of the opening times of pharmacies
February 2013	Decree-Law 34/2013	Demands an annual revision of the set of countries use for reference pricing, in order to ensure downward trend in prices.
February 2014	Decree-Law 19/2014	Further revises the margin scheme for pharmaceuticals by increasing the fixed component and decreasing the proportional component.

NOTES: The table features the most important regulations affecting pharmacy profitability during the years 2005-2015 and it is not exhaustive.

References

C.M. Gibson-Davis and M.E. Foster. A cautionary tale: Using propensity scores to estimate the effect of food stamps on food insecurity. *Social Service Review*, 80(1):93–126, 2006. doi: https://doi.org/10.1086/499416.

J.J. Heckman, H. Ichimura, and P.E. Todd. Matching as an econometric evaluation estimator: Evidence from evaluating a job training programme. *The Review of Economic Studies*, 64 (4):605–654, 1997. doi: https://doi.org/10.2307/2971733.

M. Lechner. The estimation of causal effects by difference-in-difference methods. *Foundations and Trends in Econometrics*, 4(3):165 – 224, 2010. doi: https://doi.org/10.1561/0800000014.

S.C. Salop. Monopolistic competition with outside goods. *The Bell Journal of Economics*, 10 (1):141 – 156, 1979. doi: https://doi.org/10.2307/3003323.

J.A. Smith and P.E. Todd. Does matching overcome LaLonde's critique of nonexperimental estimators? *Journal of Econometrics*, 125(1):305 – 353, 2005. doi: https://doi.org/10.1016/j.jeconom.2004.04.011.
